# AI-Driven Tumor Characterization and Histological Subtype Classification in Lung Cancer Using CT Imaging

**DOI:** 10.3390/diagnostics16172805

**Published:** 2026-08-31

**Authors:** Mohammad Shorfuzzaman, Abdullah Iftikhar, Shaheryar Najam, Jasem Almotiri, Abdullah Fawaz Aljulayfi, Dina Abdulaziz AlHammadi, Ahmad Jalal

**Affiliations:** 1 Department of Software Engineering, College of Engineering and Advanced Computing, Alfaisal University, Riyadh 11533, Saudi Arabia; 2 Department of Electrical and Computer Engineering, Riphah International University, Islamabad 44000, Pakistan; abdullahiftikhar412@gmail.com; 3 Department of Electrical Engineering, Bahria University, H-11, Islamabad 44000, Pakistan; shaheryarnajam.h11@bahria.edu.pk; 4 Department of Computer Science, College of Computers and Information Technology, Taif University, Taif 21974, Saudi Arabia; j.jasem@tu.edu.sa; 5 Department of Software Engineering, College of Computer Engineering and Sciences, Prince Sattam bin Abdulaziz University, Al-Kharj 16273, Saudi Arabia; a.aljulayfi@psau.edu.sa; 6 Department of Information Systems, College of Computer and Information Sciences, Princess Nourah bint Abdulrahman University, P.O. Box 84428, Riyadh 11671, Saudi Arabia; daalhammadi@pnu.edu.sa; 7 Department of Computer Science, Air University, Islamabad 44000, Pakistan; 8 Department of Computer Science and Engineering, College of Informatics, Korea University, Seoul 02841, Republic of Korea

**Keywords:** artificial intelligence, diagnostic AI, lung cancer, Computed Tomography (CT), medical imaging, radiomics, deep learning, MedSAM, tumor segmentation, histological subtype classification, clinical decision support

## Abstract

**Background/Objectives:** Lung cancer is still one of the top cancer mortality causes around the world, and there is a need for an accurate and clinically reliable diagnostic tool. While Computed Tomography (CT) imaging is very useful for evaluation of pulmonary nodules and tumor morphology, its interpretation is complicated by inter-patient variability, imaging artifacts, low tissue contrast, and tumor heterogeneity. Although Computer-Aided Diagnosis (CAD) systems have enhanced the diagnostic process, handcrafted feature-based approaches often fail to capture complex tumor characteristics, and numerous deep learning systems lack clinical interpretability. To tackle these challenges, this study suggests a unified diagnostic approach to characterize the tumor comprehensively. **Methods:** Lung window intensity clipping and the MedSAM foundation model are used to segment the tumor regions. After segmentation, handcrafted texture, shape, morphology and keypoint features are extracted in addition to deep features extracted by ResNet50. Particle Swarm Optimization (PSO) is used to select and refine the features, followed by an LSTM network that learns the sequential relationships among features for histological subtype classification. **Results:** It was observed that the proposed approach outperformed the benchmark approaches by attaining a higher accuracy of 93.70% and 94.70% on the Lung-PET-CT-Dx and LIDC-IDRI datasets, respectively. The ablation analysis supports the contribution of each module, clearly showing the progressive improvement of the overall classification performance obtained by integrating the complementary modules. **Conclusions:** The proposed framework effectively incorporated MedSAM-based tumor segmentation, radiomic feature analysis, and deep feature representation and sequential dependency modeling all in a single diagnostic workflow for lung cancer evaluation and diagnosis. These results prove its feasibility for explainable computer-aided diagnosis and decision support for lung cancer evaluation.

## 1. Introduction

In 2022, it is estimated that 2.48 million new cases of lung cancer and 1.82 million deaths from the disease will occur worldwide, making it the main cause of cancer death. Demographic changes, extended periods of tobacco exposure, and environmental factors continue to cause an escalating toll of diseases worldwide, of which smoking is the leading cause, accounting for almost 85% of cases [1]. Proper and timely diagnosis will make a difference in treating patients, improve patient outcomes, and aid in precision oncology. As a result, medical imaging is now an integral part of the diagnostic process and plays a crucial role in the management of lung cancer. Chest X-ray is one of the imaging modalities used and is widely available and inexpensive, but it has low spatial resolution and also suffers from overlapping anatomical structures, which may hinder the ability to detect smaller pulmonary nodules.

Functional imaging with Positron Emission Tomography (PET) is useful for staging and monitoring cancer therapy; however, the anatomical information obtained is limited and is often combined with structural imaging. In comparison, CT offers high-resolution cross-sectional imaging of pulmonary nodules, tumor morphology and surrounding anatomic structures, which is the preferred imaging modality for lung cancer screening, diagnosis, and treatment planning [2]. CT is a fundamental part of an artificial intelligence (AI) driven diagnostic system and CAD framework due to its outstanding spatial resolution and volumetric imaging. However, manual readings of CT scans are still time-consuming and prone to inter-observer variability. Even though significant progress has been made in the ability to accurately and consistently diagnose these lesions, tumor heterogeneity, lesion boundaries, low contrast between the pathological tissue and the normal tissue, motion artifacts, and anatomical complexity remain important challenges [3,4].

AI and machine learning (ML) are revolutionizing diagnostic medicine by providing computational methods for analyzing complex medical imaging data, facilitating more precise disease characterization, and informing clinical decision-making [5]. Initial CAD systems were based on a set of manually derived radiomic descriptors that characterize tumor texture, intensity distributions, shape features and morphological patterns in a meaningful way [6,7]. These radiomic markers are clinically relevant and have proven to be quite useful in diagnostic evaluation and risk stratification. Handcrafted feature engineering, however, has a tendency to miss the complex spatial hierarchies, contextual relationships and heterogeneous imaging patterns that exist in different patient populations and acquisition protocols [8].

The paradigm of deep learning is becoming a powerful tool for medical image analysis, as it directly learns the hierarchical features of the images. Convolutional Neural Networks (CNNs) have also recently been successful for the detection, classification, segmentation, and prediction of diagnostic information in tumor data [9,10]. Architectures like Residual Networks can capture more complex visual features and context dependencies that are hard to encode by handcrafted descriptors. However, many deep learning models remain “black-box” meaning that they are not interpretable and therefore less trusted in the clinical setting or even accepted for use. In this context, the use of frameworks that combine interpretable imaging biomarkers with powerful deep feature representations for enhanced diagnostic accuracy and clinical transparency is emerging.

In response to these challenges, this study introduces a hybrid diagnostic framework for the characterization of lung cancer and the classification of its histological types. The proposed framework aims to combine the complementary information provided by the structural, textural, shape and semantic features of Computed Tomography (CT) imaging to map the tumor by segmentation using MedSAM, extract radiomic features, learn multi-view features via deep learning, and fuse the features using a hybrid approach. To ensure optimal selection of the most discriminative fused features before sequential classification, an optimization stage is implemented, and to enhance the interpretability of the model, Gradient-weighted Class Activation Mapping (Grad-CAM) visualization and feature analysis are added. The proposed system integrates segmentation, handcrafted and deep feature representations, feature optimization of features, and classification into a single framework with the goal of enhancing automatic lung cancer diagnosis and facilitating accurate clinical decision-making.

The primary contributions of this work are summarized as follows:

- AI-Driven Hybrid Diagnostic Framework: A unified diagnostic framework is proposed that combines handcrafted radiomic biomarkers, including texture, structural, and geometric descriptors, with deep semantic representations extracted using ResNet-50. This integration enables comprehensive, clinically meaningful, and interpretable tumor characterization from CT images.
- Foundation Model-Based Tumor Segmentation: A prompt-guided MedSAM segmentation strategy is incorporated to accurately localize tumor regions using bounding-box supervision. The resulting ROI-aware analysis focuses feature extraction on clinically relevant pathological tissues while reducing background interference and improving biomarker reliability.
- Multi-Level Radiomic Biomarker Characterization: The framework integrates complementary radiomic descriptors for enhanced tumor phenotyping and histological subtype differentiation:
  1. Structural features: ORB and Harris keypoints for local structural representation.
  2. Shape descriptors: Hu invariant moments for global tumor geometry characterization.
  3. Texture features: GLCM and LBP for modeling spatial intensity distributions and micro-textural patterns.
  4. This multi-level radiomic analysis provides a comprehensive representation of tumor heterogeneity and improves diagnostic discrimination among lung cancer subtypes.
- Optimized Feature Selection and Sequential Diagnostic Learning: An integrated optimization framework combining PSO and Long Short-Term Memory (LSTM) networks is introduced. PSO identifies the most discriminative fused features by reducing redundancy in high-dimensional feature spaces, while LSTM models inter-slice dependencies within CT volumes to capture spatial continuity and disease progression patterns, thereby improving diagnostic accuracy and robustness.
- Comprehensive Validation and Clinical Relevance Assessment: Extensive ablation studies validate the effectiveness of each framework component and demonstrate that deep feature representations, radiomic biomarkers, segmentation-guided analysis, feature fusion, and optimization collectively contribute to improved classification performance, diagnostic reliability, and generalization capability across diverse imaging scenarios.

The remainder of this paper is organized as follows. Section 2 reviews recent advances in AI-driven lung cancer diagnosis, medical image segmentation, radiomics, and classification techniques. Section 3 presents the proposed methodology, including preprocessing, data augmentation, MedSAM-based segmentation, feature extraction, feature fusion, optimization, and classification. Section 4 discusses experimental results, comparative evaluations, explainability analysis, and computational analysis. Furthermore, a comprehensive ablation study is performed to systematically evaluate the individual contribution of each component of the proposed framework, including preprocessing, deep feature extraction, structural and textural radiomic descriptors, shape analysis, and feature optimization. Finally, Section 5 concludes the study and outlines future research directions.

## 2. Related Work

One of the deadliest malignancies in the world, lung cancer has early detection as a crucial element in enhancing patient survival Reck et al. [11]. CT is the standard screening and diagnosis imaging modality which provides details of the morphology of tumors and fine details of the pulmonary nodules. The low-contrast limits and small size of lesions of the CT scans complicate the manual interpretation of the scan to allow a rapid process of diagnosis and treatment planning Wang et al. [12] and Kashyap et al. [13].

A. Machine Learning Approaches

CAD systems were created to enhance detection and evaluation and help radiologists by identifying suspicious areas automatically and providing quantitative information. The initial CAD pipelines were based on handcrafted radiomics features that included intensity, texture, and shape. These characteristics allowed characterization and classification of tumors in an interpretable manner Leng et al. [6], Alsallal et al. [14].

Dunn et al. [15] directly tested radiomics using classical machine learning classifiers on the Lung-PET-CT-Dx dataset, which yielded texture and shape features to distinguish between lung cancer subtypes. Despite their utility, handcrafted features themselves frequently failed in terms of dealing with complex tumor heterogeneity and not generalizing to different acquisition protocols [15]. This was the impetus to move towards deep learning and hybrid methods to better analyze lung tumors.

B. Deep Learning Approaches

Deep learning has made a large breakthrough in lung cancer detection and classification on CT images. CNNs and their derivatives have been shown to perform better in learning hierarchical features directly on imaging data, without the need to specify hierarchical features [16]. Loja et al. [17] have introduced a multi-class classification model based on deep learning and PET images in the Lung-PET-CT-Dx dataset but obtained significantly low accuracy in the classification of histological subtype. In a similar manner, Chowdhury et al. [18] proposed an anatomy-sensitive hybrid deep learning model, which uses spatial context to detect and classify objects better than the same dataset with only the model. Transformer-based networks have also been discussed in the recent literature Barbouchi et al. [19] and improved YOLO architectures Zeng et al. [20] to be effective in nodule detection.

Despite this great advancement, there are a number of issues encountered in the field of lung tumor analysis, especially small lesions, low-contrast boundaries and inter-slice variations. Even high-dimensional fused features may bring about redundancy and overfitting across different CT protocols Wang et al. [16]. These drawbacks are increasingly being addressed using hybrid radiomics-deep pipelines which are more interpretable, robust and capture sequential tumor features. On the whole, these combined efforts open up the way to support computer-aided diagnosis systems.

## 3. System Methodology

**Figure 1:**
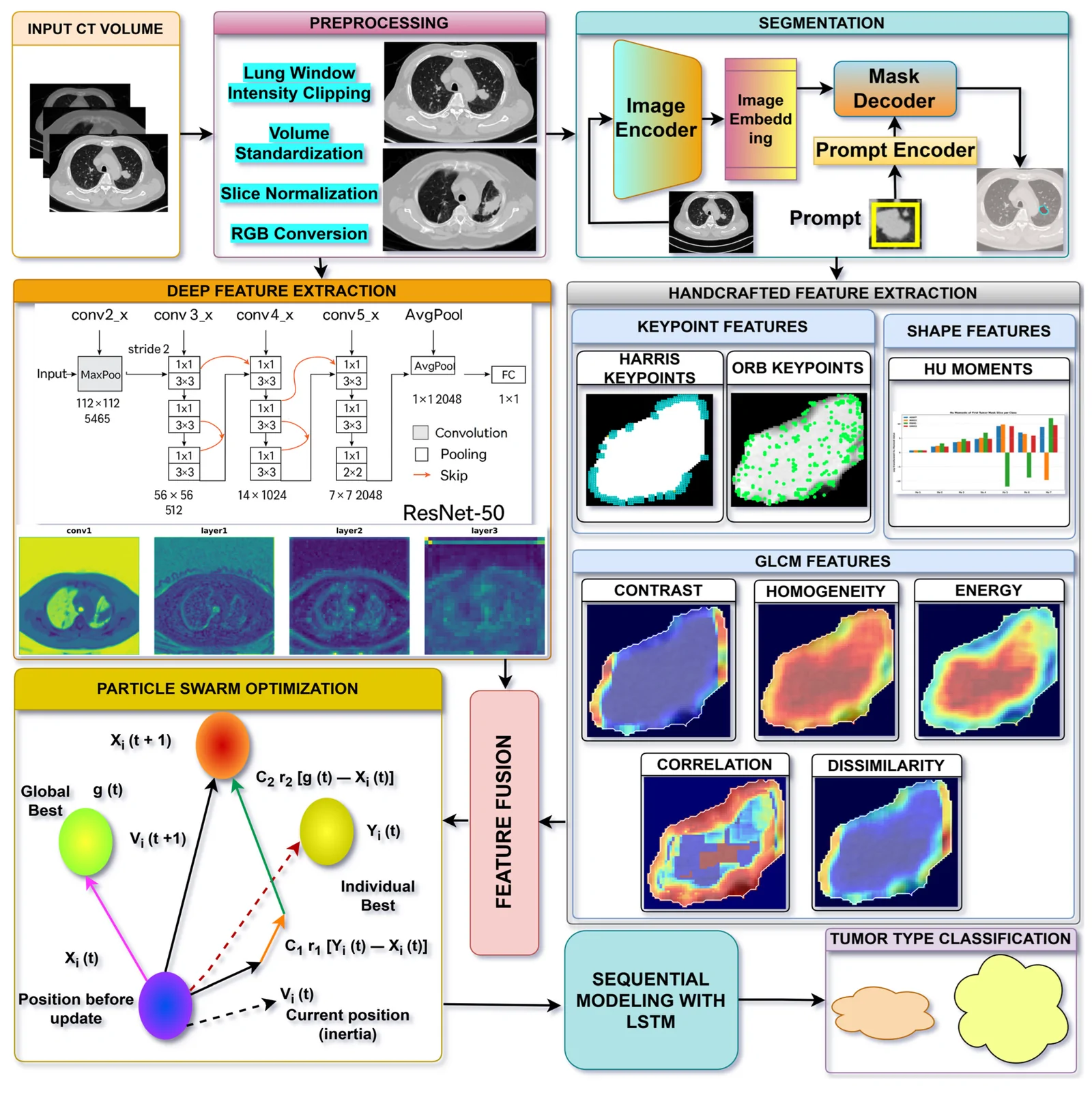
Block diagram of the proposed hybrid framework for lung cancer subtype classification.

The proposed study proposes a hybrid approach for lung cancer characterization and subclassification of histological subtypes from CT images. The framework consists of CT image preprocessing, MedSAM for tumor segmentation, radiomic feature extraction, deep feature learning, feature fusion, optimization, and sequential classification. Data in the CT volumes are first normalized for image consistency, and data augmentation is used to tackle class imbalance and improve the representation of minority tumor types. Next, MedSAM is used to precisely identify tumor ROIs and assist with focused analysis of pathological tissues. The segmented tumors are used to extract complementary radiomic features to represent interpretable texture, shape, and structural properties, while a pre-trained ResNet-50 network is used to learn high-level semantic representations from CT slices. This results in fused and optimized features for maximum discriminative diagnostic information. Finally, optimized feature sequences are fed into an LSTM network to model inter-slice dependencies and perform histological subtype classification. To validate the effectiveness of the proposed framework, a comprehensive ablation study was conducted by systematically removing individual components, including preprocessing, deep feature extraction, structural and textural radiomic descriptors, shape analysis, and feature optimization. This analysis quantifies the contribution of each module to the overall classification performance and demonstrates the importance of the proposed hybrid architecture. The proposed framework combines medical image segmentation, radiomics, and deep learning to facilitate automated lung cancer diagnosis and improve clinical decision-making. The overall architecture of the proposed system is illustrated in Figure 1, while the following subsection describes the preprocessing stage applied before segmentation and feature extraction.

### 3.1. CT Image Preprocessing

CT scans obtained from different scanners often exhibit variations in intensity distribution and imaging parameters. To minimize this variation and guarantee consistency across all subjects, a preprocessing step is used to normalize CT intensities before feature extraction.

#### 3.1.1. Lung Window Intensity Clipping

CT images represent tissue density in Hounsfield Units (HU), where lung parenchyma and soft tissues occupy a specific intensity range. To focus the analysis on relevant lung structures and suppress extreme intensity values associated with dense tissues such as bone, intensity clipping is performed using a standard lung window. Let I(x,y,z) denote the original CT intensity at voxel location $\left(x,y,z\right)$. The preprocessed intensity $I_{p}(x,y,z)$ is obtained through clipping within the range $\left[-1000,400\right]$ HU as given in Equation (1).

(1) $$I_{p}\left(x,y,z\right)=min\left(400,max\left(-1000,I\left(x,y,z\right)\right)\right)$$

This transformation preserves the intensity distribution of lung tissue while limiting the influence of outlier values that may adversely affect feature computation (Oh et al., 2025 [7]):

#### 3.1.2. Volume Standardization

After intensity normalization, each CT volume is represented as a three-dimensional array as shown in Equation (2).

(2) $$V\in R^{H\times W\times S}$$

where H and W denote the in-plane spatial dimensions and S represents the number of slices in the scan. The associated voxel spacing information is preserved for each subject to maintain the original spatial characteristics of the imaging data. The resulting preprocessed CT volumes serve as the standardized input for subsequent tumor region extraction and feature computation stages.

#### 3.1.3. Slice Preparation

Each CT slice is first normalized and converted into a format compatible with the MedSAM model. Since MedSAM expects three-channel inputs, the single-channel CT slice is replicated across three channels after intensity normalization. Let $I_{s}(x,y)$ denote the intensity of slice s. The normalized image $I_{s}^{\prime }(x,y)$ is computed as given in Equation (3).

(3) $$I_{s}^{\prime }\left(x,y\right)=\frac{I_{s}\left(x,y\right)-min\left(I_{s}\right)}{max\left(I_{s}\right)-min\left(I_{s}\right)}$$

The normalized slice is then scaled to the range $\left[0,255\right]$ and converted to an RGB representation by duplicating the intensity channel.

#### 3.1.4. Data Augmentation

The dataset utilized in this study has a significant class imbalance, with Lung Adenocarcinoma being overrepresented compared to minority classes like Small Cell Carcinoma. This skew may skew the learning process, leading the model to be biased towards majority classes and underperform on less-represented categories. To solve this problem, data augmentation is specifically used on the minority classes to enhance their effective sample size and the overall balance of classes. After preprocessing, augmentation is done at the slice level, and label-preserving transformations, including rotation, horizontal and vertical flipping, scaling and small intensity changes, are used. These procedures bring about controlled variability without affecting the anatomical and structural integrity of lung tissues and tumor areas. The augmented samples are added to the training set, which allows the model to learn more robust and generalizable features and eventually leads to better performance in classifying all subtypes of lung cancer.

### 3.2. Tumor Region Segmentation Using MedSAM

Another key characteristic for AI-based diagnostics is tumor delineation, which directly influences the reliability of extracted imaging biomarkers and subsequent classification. Conventional medical image segmentation methods, including U-Net-based and other supervised architectures, generally require pixel-level ground-truth masks and often involve dataset-specific training or fine-tuning, which can be challenging when such annotations are unavailable. In this context, foundation models offer an alternative by enabling segmentation with reduced dependence on extensive manual supervision. The Segment Anything Model (SAM) introduced a prompt-driven segmentation paradigm with strong generalization across diverse visual tasks, while its medical adaptation, MedSAM, was developed to address the characteristics of medical images and support prompt-guided lesion segmentation [21]. In the proposed framework, MedSAM is employed to generate tumor regions from available spatial prompts, thereby shifting the segmentation process from fully supervised pixel-level annotation toward a prompt-based weakly supervised setting. This approach avoids the need to develop and fine-tune a separate segmentation model using manually annotated masks, while providing a more transferable mechanism for tumor delineation and subsequent ROI-based feature extraction across heterogeneous CT datasets. 

For accurate delineation of tumor regions to enable reliable feature extraction, in this study, tumor segmentation is performed using MedSAM, a segmentation framework adapted for medical imaging tasks [21]. Let the preprocessed CT volume be represented as defined in Equation (4).

(4) $$V\in R^{S\times H\times W}$$

where S denotes the number of slices and H×W represents the spatial resolution of each slice. For a given slice s, a bounding region indicating the approximate tumor location is defined as given in Equation (5).

(5) $$B_{s}=\left(x_{min},y_{min},x_{max},y_{max}\right)$$

**Figure 2:**
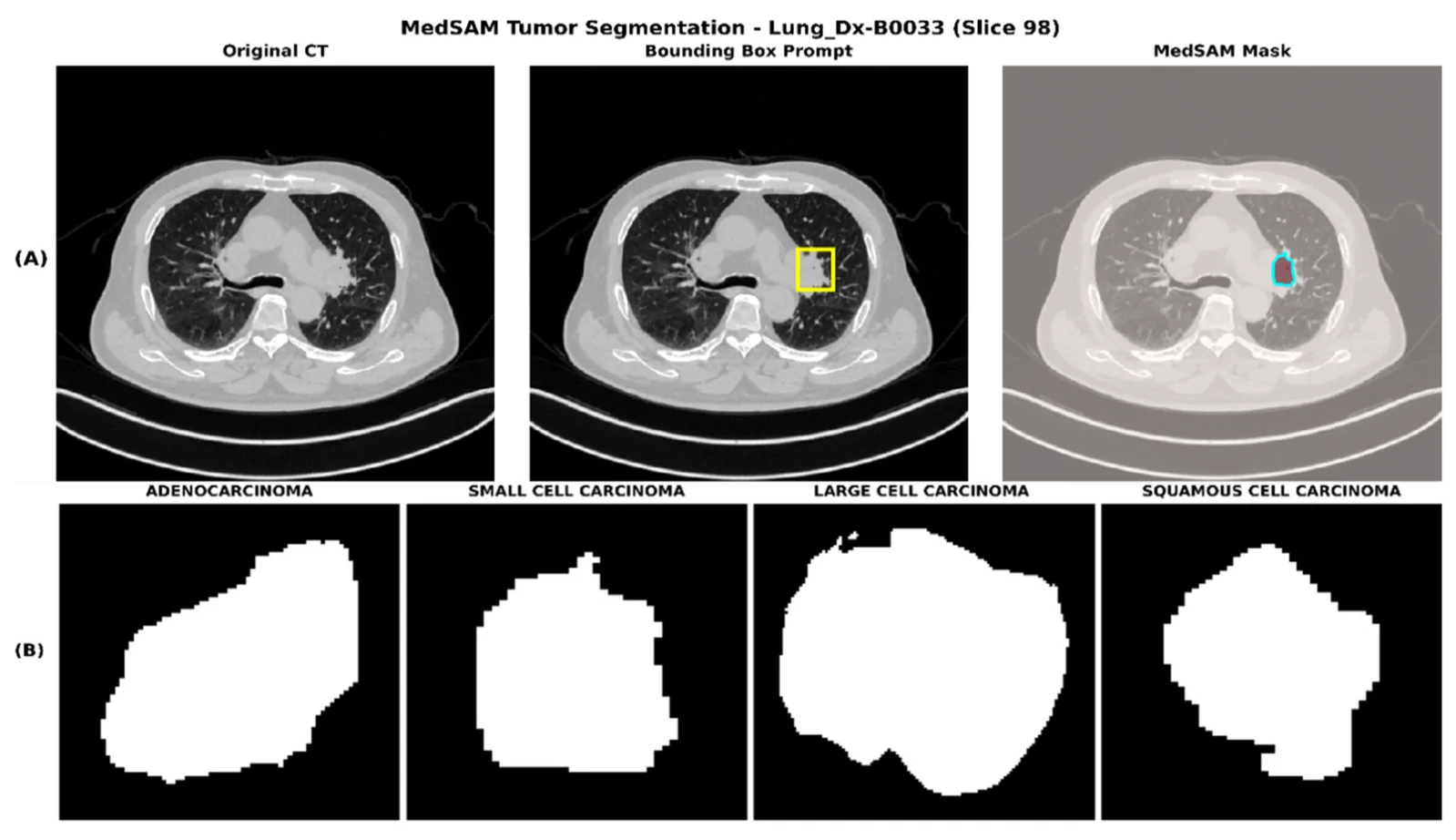
Representative stages of tumor segmentation: (**A**) original CT slice, bounding box overlay, MedSAM-generated tumor mask; (**B**) class-wise predicted masks.

Given the slice $I_{s}$ and the corresponding region $B_{s}$, MedSAM predicts a binary tumor mask as computed by Equation (6).

(6) $$M_{s}=f_{MedSAM}\left(I_{s},B_{s}\right)$$

where $f_{\mathrm{MedSAM}}$ denotes the segmentation network and

(7) $$M_{s}\in \{0,1{\}}^{H\times W}$$

represents the predicted tumor mask for slice s as shown in Equation (7). MedSAM was deployed in a zero-shot manner, performing inference without any retraining or fine-tuning, with the resulting masks serving as regions of interest for subsequent feature extraction and classification. The MedSAM model was originally trained using a segmentation objective that combines pixel-wise classification and region overlap. The Dice loss used to measure segmentation accuracy is defined as Equation (8).

(8) $$L_{Dice}=1-\frac{2\left|P\cap G\right|}{\left|P\right|+\left|G\right|}$$

where P is the predicted mask and G denotes the reference segmentation. Consequently, segmentation metrics such as Dice and IoU were not evaluated in this study. When multiple bounding regions exist in a slice, the resulting masks are merged using a pixel-wise maximum operation as given in Equation (9).

(9) $$M_{s}\left(x,y\right)={max}_{k}M_{s,k}\left(x,y\right)$$

where $M_{s,k}$ represents the mask generated for the $k^{th}$ bounding region. Following the segmentation, each annotated slice in the CT volume is associated with a corresponding tumor mask $M_{s}$. Since lung tumors may span multiple adjacent slices, all segmented slices are retained to preserve the volumetric tumor structure. The resulting set of tumor masks across slices forms a sequential representation of the tumor region, which is subsequently utilized for feature extraction and temporal modeling. Representative stages of the tumor segmentation process using MedSAM are shown in Figure 2.

### 3.3. Structural and Geometric Feature Analysis Using Keypoint Detection and Invariant Moments

To comprehensively characterize tumor regions, both local structural variations and global geometric properties are analyzed. This is achieved by integrating keypoint-based structural analysis with shape-based feature extraction using invariant moments. Whereas keypoint detection reveals fine-grained structural irregularities within and at the edges of tumors, invariant moments give a concise description of the overall tumor morphology. The combination of these complementary methods allows a strong description of tumor complexity. To study structural complexity in tumor regions, keypoint detection techniques are used on the tumor mask. Two complementary detectors are utilized.

#### 3.3.1. ORB (Oriented FAST and Rotated BRIEF)

ORB is a computationally efficient keypoint detector and descriptor that combines the FAST corner detector with the BRIEF descriptor, extended to achieve rotation and scale invariance. Given an input image, ORB first applies FAST to detect candidate keypoints by comparing the intensity of a candidate pixel against a circular ring of neighboring pixels. A pixel is classified as a keypoint if a sufficient number of contiguous neighbors are either brighter or darker than the candidate by a threshold, as defined in Equation (10).

(10) $$I_{p}-I_{i}>\epsilon \;\mathrm{or}\;I_{i}-I_{p}>\epsilon$$

where $I_{p}$ is the intensity of the candidate pixel, $I_{i}$ is the intensity of the i-th neighbor on the Bresenham circle, and ϵ is the contrast threshold. To achieve rotation invariance, ORB computes an orientation angle for each keypoint using intensity-weighted centroid moments and steers the BRIEF descriptor accordingly. The resulting binary descriptor is computed as a set of pairwise intensity comparisons in a rotated patch, defined in Equation (11).

(11) $$\tau \left(p;x,y\right)=\left\{\begin{array}{ll} 1 & p\left(x\right)<p\left(y\right)\\ 0 & \mathrm{otherwise} \end{array}\right.$$

where p(x) and p(y) are the smoothed intensities at locations x and y within the oriented patch. The final descriptor is a compact binary string formed by n such comparisons, enabling fast matching via Hamming distance. In this work, ORB was applied to ROI-aware CLAHE-enhanced tumor regions to detect structurally significant keypoints along tumor margins and boundary transition zones in CT images.

#### 3.3.2. Harris Corner Detector

In contrast, the Harris detector identifies corner-like structures by analyzing local intensity gradients. The Harris response function is defined as Equation (12).

(12) $$R=det\left(H\right)-k{\left(trace\left(H\right)\right)}^{2}$$

where the second-moment matrix is shown in Equation (13).

(13) $$H=\left[\begin{array}{cc} I_{x}^{2} & I_{x}I_{y}\\ I_{x}I_{y} & I_{y}^{2} \end{array}\right]$$

represents the second-moment matrix derived from image gradients $I_{x}$ and $I_{y}$, and k is a sensitivity parameter. The points that have high response values are associated with strong corner structures. The identified keypoints reveal structural differences on tumor edges and within the tumor regions that indicate the complexity of the tumor.

#### 3.3.3. Hu Moments

Besides the local structure-related characteristics, global geometric characteristics of tumor shape could be derived with the help of Hu invariant moments. These properties are obtained using the binary tumor mask, and they are translation-, rotation-, and scaling-invariant. In the case of a binary tumor mask M(x,y) the spatial moments are given by Equation (14).

(14) $$m_{pq}=\sum_{x}\sum_{y}x^{p}y^{q}M\left(x,y\right)$$

From these, the centroid of the tumor region is computed as in Equation (15).

(15) $$\bar{x}=\frac{m_{10}}{m_{00}},\;\bar{y}=\frac{m_{01}}{m_{00}}$$

Central moments are then calculated as in Equation (16).

(16) $$\mu_{pq}=\sum_{x}\sum_{y}{\left(x-\bar{x}\right)}^{p}{\left(y-\bar{y}\right)}^{q}M\left(x,y\right)$$

Normalized central moments are obtained as in Equation (17).

(17) $$\eta_{pq}=\frac{\mu_{pq}}{\mu_{00}^{1+\frac{p+q}{2}}}$$

From the normalized moments, seven Hu invariant moments are composed of $H_{1},H_{2},\ldots ,H_{7}$ are computed to characterize the tumor shape. Since these values can range several orders of magnitude, a logarithmic transformation is applied as in Equation (18).

(18) $$H_{i}^{\prime }=-sign\left(H_{i}\right){log}_{10}\left|H_{i}\right|$$

**Figure 3:**
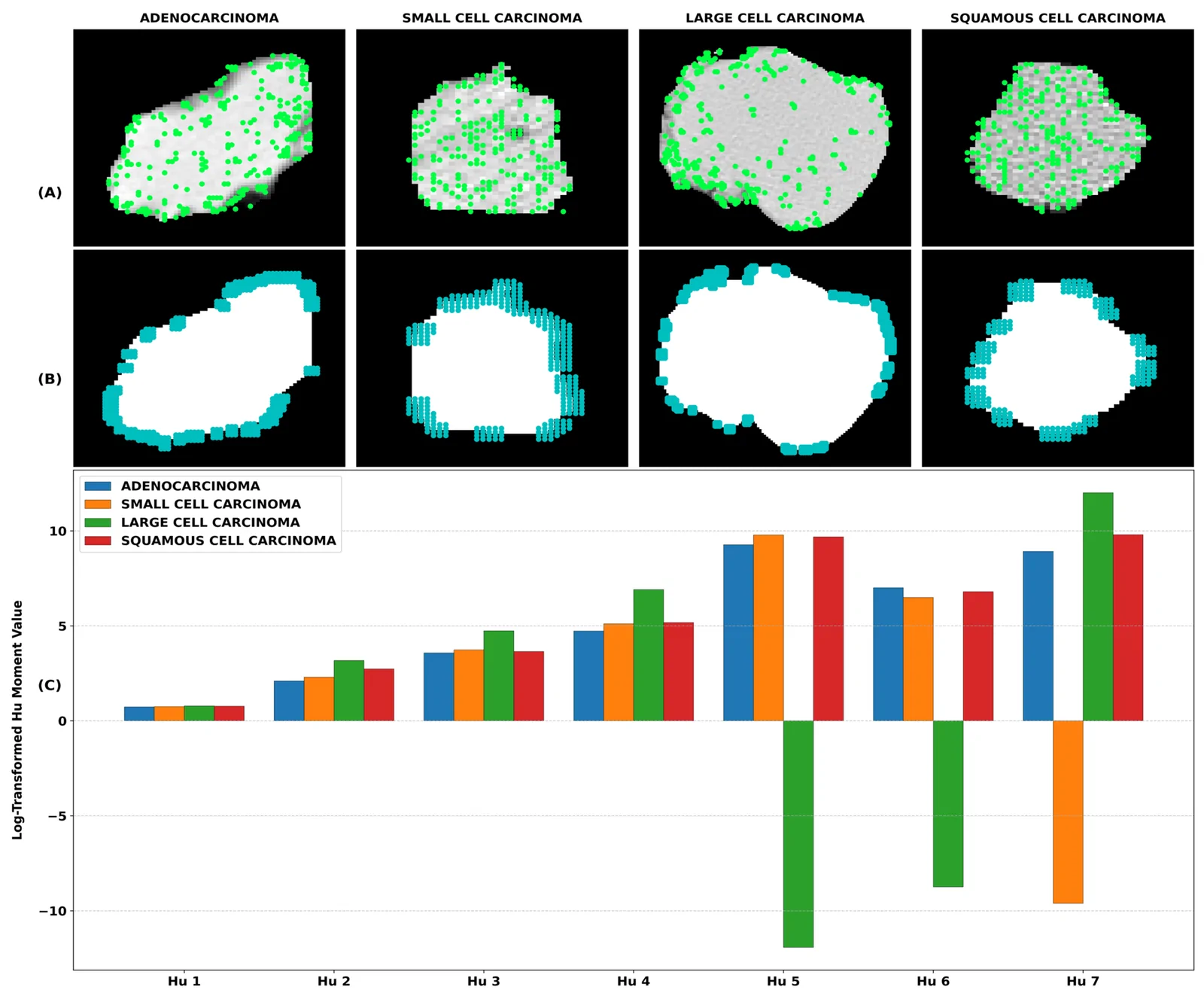
Class-wise Structural and Geometric Features (**A**) ORB (**B**) Harris Corner Detector (**C**) Log-transformed Hu moments.

These features provide a compact representation of tumor geometry which complements the texture-based descriptors. The class-wise keypoint detection results along with Hu moment features for different lung cancer subtypes are presented in Figure 3.

### 3.4. Texture Feature Extraction Using Gray-Level Co-Occurrence Matrix (GLCM)

Texture features are computed using the GLCM to characterize spatial relationships between neighboring pixels within the tumor region [7]. Prior to GLCM computation, CT intensities are quantized into L discrete gray levels to reduce computational complexity. Let the quantized image be denoted as Q (x, y). The GLCM P (i, j, d, θ) represents the probability of observing pixel values i and j separated by distance d at orientation θ. In this study, GLCM is computed using d = 1, θ = 0° over a sliding window centered at each tumor pixel. From the GLCM, many texture descriptors are computed.

#### 3.4.1. Contrast

The contrast attribute determines the intensity variation by focusing on greater gray-level differences among neighboring pixels. It is due to this characteristic that the contrast attribute proves to be more suitable for characterizing tumor areas, where abrupt intensity changes are prominent, as defined by Equation (19).

(19) $$\mathrm{Contrast}=\Sigma_{i,j}{\left(i-j\right)}^{2}P\left(i,j\right)$$

#### 3.4.2. Homogeneity

Homogeneity measures the similarity between neighboring pixel intensities by giving higher weight to elements near the diagonal of the GLCM. Regions with smoother and more uniform textures naturally attain higher homogeneity values, capturing the local structural regularity of the tumor, as shown in Equation (20).

(20) $$\mathrm{Homogeneity}=\Sigma_{i,j}\frac{P\left(i,j\right)}{1+\left|i-j\right|}$$

#### 3.4.3. Energy

Energy is representative of the overall textural uniformity within the tumor region. Areas having repetitive or highly ordered intensity patterns will give higher energy values, while more complex textures produce lower values. This relationship is formalized in Equation (21).

(21) $$\mathrm{Energy}=\sum_{i,j}P{\left(i,j\right)}^{2}$$

#### 3.4.4. Correlation

Two consecutive gray level values are said to have a linear dependency between their values, called correlation, which gives us an indication of the stability of pixel intensities with regard to one located on the opposite side of the tumor. Structured textures exhibit stronger correlations, which may be quantified as expressed in Equation (22).

(22) $$\mathrm{Correlation}=\sum_{i,j}\frac{\left(i-\mu_{i}\right)\left(j-\mu_{j}\right)P\left(i,j\right)}{\sigma_{i}\sigma_{j}}$$

#### 3.4.5. Dissimilarity

Dissimilarity calculates the absolute differences between adjacent gray levels, offering a reliable indicator of texture variation. Different from contrast, it is less influenced by large intensity differences while still highlighting moderate heterogeneity, as depicted in Equation (23).

(23) $$\mathrm{Dissimilarity}=\Sigma_{i,j}\left|i-j\right|P\left(i,j\right)$$

**Figure 4:**
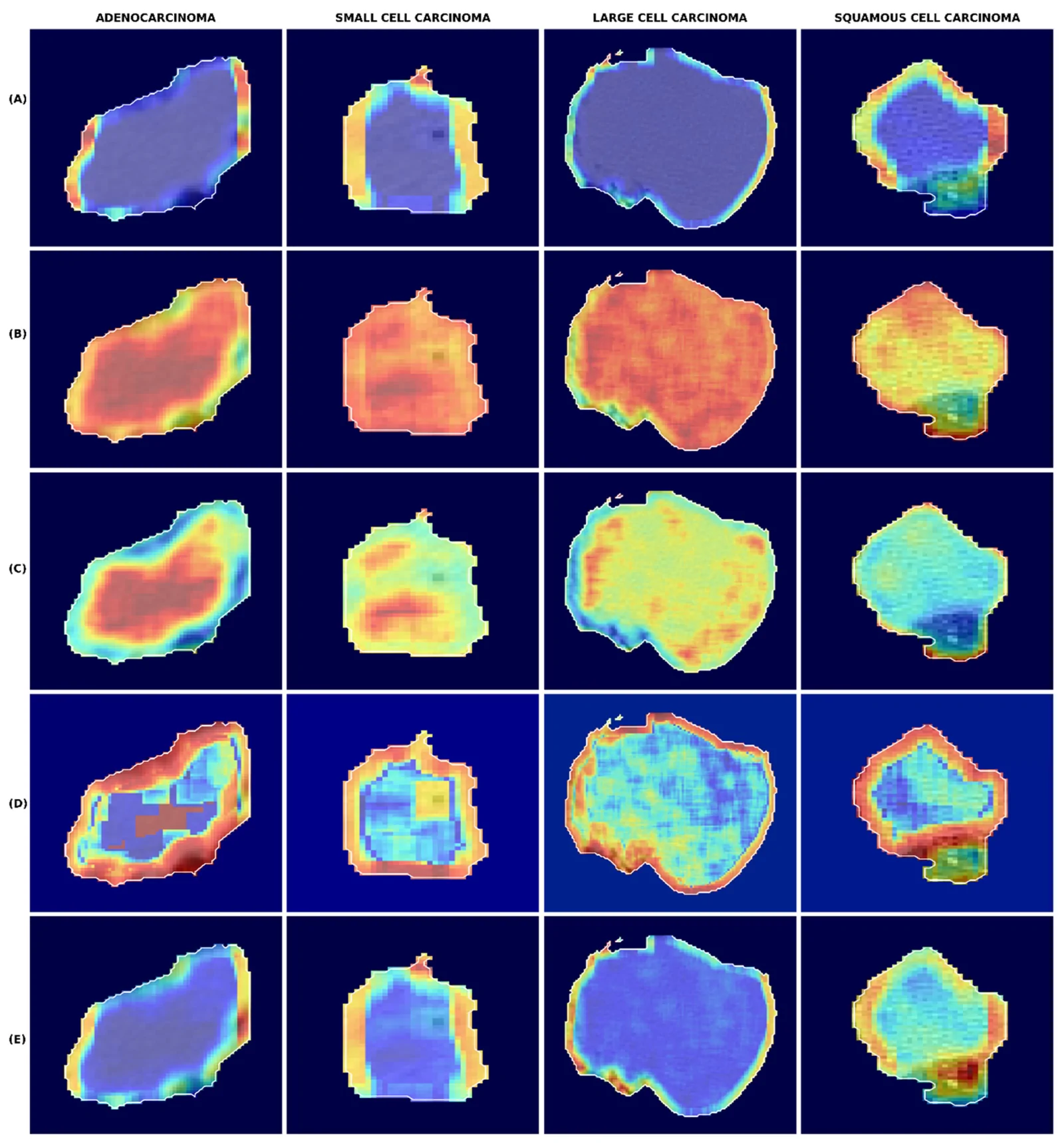
Class-wise GLCM texture features: (**A**) Contrast, (**B**) Homogeneity, (**C**) Energy, (**D**) Correlation, and (**E**) Dissimilarity.

These attributes are calculated locally through moving windows in the tumor regions to generate GLCM feature maps indicating the spatial variance of texture in the tumor. GLCM texture attributes for various types of lung cancer are shown in Figure 4.

### 3.5. Texture Representation Using Local Binary Patterns (LBP)

Other than GLCM descriptors, LBP features are employed to capture fine-scale micro-texture variations within tumor tissue [8]. LBP feature of each pixel p involves a comparison between its value and its neighboring values around a circular radius of R. These neighboring pixels amount to P pixels. LBP code is presented in Equation (24).

(24) $$LBP_{\left(P,R\right)}=\sum_{n=0}^{P-1}s\left(g_{n}-g_{c}\right)2^{n}$$

where

(25) $$s\left(x\right)=\left\{\begin{array}{ll} 1 & x\geq 0\\ 0 & x<0 \end{array}\right.$$

**Figure 5:**
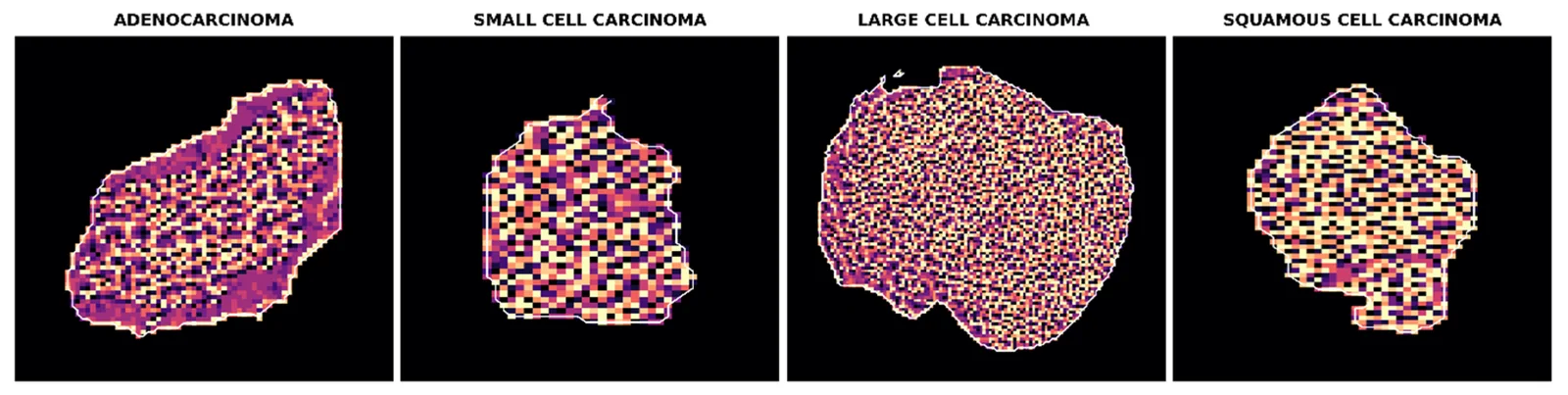
Class-wise LBP texture maps for lung cancer subtypes.

In this formulation, $g_{c}$ corresponds to the intensity of the central pixel, while $g_{n}$ denotes the intensities of its neighboring pixels. The parameters in our implementation were set to P = 8 and R = 1, and the uniform variant of LBP was used to reduce the size of the pattern space whilst preserving discriminative texture features. To make sure that the features extracted only capture tumor-specific features, LBP was calculated directly in the segmented tumor area and thus reduces the influence of other lung tissue. The resulting LBP maps emphasize local fine structures, such as edges, spot-like objects and homogeneous areas of the tumor. Figure 5 shows representative LBP texture maps of various subtypes of lung cancer.

### 3.6. Deep Feature Extraction Using ResNet-50

Deep convolutional representations are also derived in addition to handcrafted radiomic features to the latter in order to capture more abstract spatial features of tumor regions [16]. To this end, a ResNet-50 model is employed as the main feature extractor, as this model has been shown to be effective in medical imaging. It is architecturally based on the concept of residual learning, helping to address the problems of degradation and vanishing gradient that are prevalent in deep networks. Instead of directly learning a mapping H(x), the model focuses on learning a residual function F(x) defined in Equation (26), allowing the network to better preserve and refine feature representations across layers.

(26) $$F\left(x\right)=H\left(x\right)-x$$

which leads to the residual formulation in Equation (27).

(27) $$H\left(x\right)=F\left(x\right)+x$$

In this case, x refers to the input feature map, and the residual connections facilitate stable gradient flow in addition to allowing the network to learn complex visual patterns. In this paper, every slice of tumor is first rescaled to the input specification of ResNet-50 and turned into three-channel format. The network is then processed and the global average pooling layer provides feature representations, yielding a feature vector $f_{\mathrm{deep}}\in R^{2048}$.

**Figure 6:**
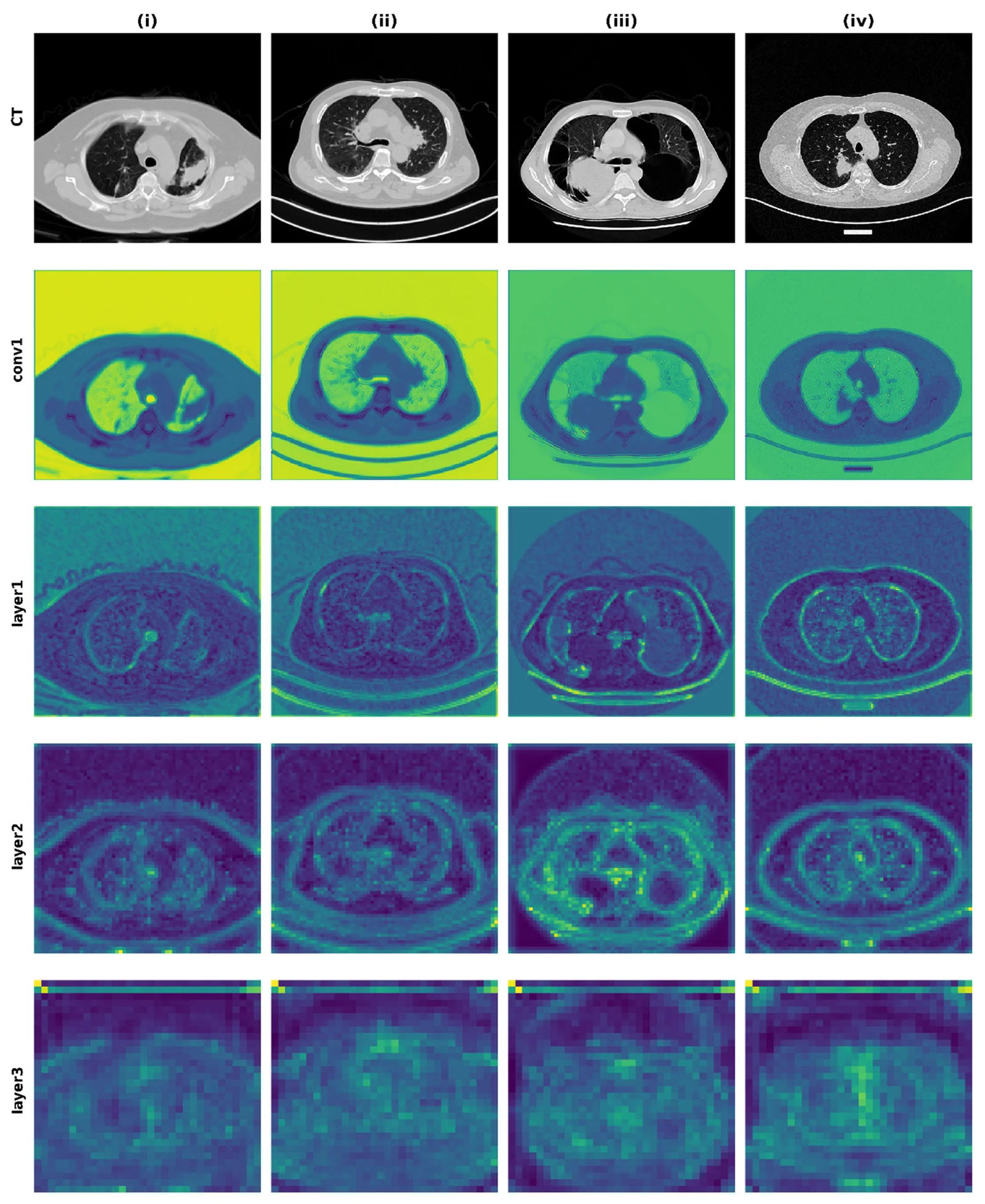
Representative CT Slices and layer activation maps from ResNet-50: (**i**) Adenocarcinoma, (**ii**) Small Cell Carcinoma, (**iii**) Large Cell Carcinoma, (**iv**) Squamous Cell Carcinoma.

These high-level structural and contextual features, which Figure 6 demonstrates, are complementary to handcrafted descriptors, especially in the description of patterns that are not readily described using the conventional radiomic techniques.

### 3.7. Hybrid Feature Fusion

A hybrid feature fusion scheme is adopted to merge handcrafted descriptors with deep learning-based representations to obtain complementary information in tumor regions in one feature space. The handcrafted features are aimed at capturing various attributes of tumor features, such as shape information based on Hu invariant moments, structure features based on keypoints identified by ORB and Harris algorithms, and texture features based on GLCM and Local Binary Patterns. Combined, these elements constitute the handcrafted feature vector, as given in Equation (28).

(28) $$f_{\mathrm{handcrafted}}=\left[f_{\mathrm{shape}},f_{\mathrm{structure}},f_{\mathrm{texture}}\right]$$

To further enrich the representation, this handcrafted vector is combined with the deep feature vector obtained from the network. The resulting fused representation unites radiomic characteristics and high-level semantics that the model has acquired, enabling a more comprehensive characterization of tumor appearance. The following combined feature vector, as defined in Equation (29), incorporates both low-level and abstract patterns, improving the overall representation capability.

(29) $$f_{\mathrm{hybrid}}=\left[f_{\mathrm{handcrafted}}\|f_{d}\right]$$

The dimensionality of each feature descriptor depends on its underlying representation and extraction strategy. ORB generates 32-dimensional binary descriptors for each detected keypoint; therefore, retaining the strongest 100 keypoints results in a 3200-dimensional feature vector. Similarly, Harris corner detection preserves the spatial coordinates of the 100 most salient corner points, producing a 200-dimensional descriptor (100 × 2 coordinates). In contrast, Hu moments, GLCM, and LBP generate compact statistical descriptors with fixed dimensions independent of the number of detected points. The ResNet-50 backbone contributes a 2048-dimensional deep feature vector obtained from the global average pooling layer. Consequently, concatenating all handcrafted and deep descriptors yields a 5470-dimensional hybrid feature vector, as summarized in Table 1.

By leveraging information from both domains, the hybrid feature vector provides a more robust characterization of tumor heterogeneity and supports improved differentiation between tumor classes [14].

### 3.8. Particle Swarm Optimization for Feature Selection

The deep convolutional features coupled together with handcrafted texture descriptors lead to a high-dimensional representation. This merged feature space has the potential to model a wide range of complementary tumor properties, but may contain redundant or less informative features, both of which may negatively affect classification outcome as well as add unnecessary computation cost. To reduce this, Particle Swarm Optimization (PSO) is used as a feature selection method to find the most pertinent subset of features.

PSO is a population-based metaheuristic optimization technique inspired by the collective behavior of bird flocks and fish schools. In the proposed framework, each particle represents a candidate subset of features encoded as a binary vector represented in Equation (30).

(30) $$p_{i}=\left[p_{i1},\ldots ,p_{id}\right],\;p_{ij}\in \{0,1\}$$

where d denotes the dimensionality of the fused feature vector and $p_{ij}=1$ indicates that the $j^{th}$ feature is selected.

During optimization, particles explore the search space by updating their velocities and positions according to Equations (31) and (32).

(31) $$v_{i}^{t+1}=wv_{i}^{t}+c_{1}r_{1}\left(pbest_{i}-x_{i}^{t}\right)+c_{2}r_{2}\left(gbest-x_{i}^{t}\right)$$

(32) $$x_{i}^{t+1}=x_{i}^{t}+v_{i}^{t+1}$$

where $v_{i}$ represents the particle velocity, $x_{i}$ denotes the particle position, w is the inertia weight controlling exploration, $c_{1}$ and $c_{2}$ are acceleration coefficients, and $r_{1},r_{2}$ are random variables sampled from a uniform distribution in $\left[0,1\right]$. The terms $pbest_{i}$ and gbest correspond to the individual best and global best solutions obtained during the optimization process. The fitness of each particle is evaluated based on classification performance while encouraging compact feature subsets. The objective function is defined in Equation (33).

(33) $$F=\alpha Acc-\beta \frac{N_{s}}{N_{f}}$$

where Acc denotes classification accuracy, $N_{s}$ is the number of selected features, $N_{f}$ represents the total number of features in the fused vector, and α and β are weighting parameters controlling the trade-off between accuracy and dimensionality reduction. The accuracy term (Acc) in Equation (33) is calculated exclusively on the training partition of the Lung-PET-CT-Dx dataset. Each particle represents a candidate binary feature subset selected from the 5470-dimensional fused feature representation. For every candidate subset, the corresponding training features are provided to an SVM classifier, and the resulting classification accuracy is used as the performance component of the PSO fitness function. The fitness function jointly considers the classification accuracy and the number of selected features, thereby encouraging PSO to identify a compact subset that retains strong discriminative capability while reducing feature redundancy. The SVM is used only as the fitness evaluator during this standalone feature-selection stage and is not used as the final classifier. After the PSO search converges, the globally selected feature subset is fixed and subsequently used to construct the input representation for the proposed LSTM classifier. Importantly, the test partition of Lung-PET-CT-Dx remains completely unseen throughout the PSO search and is not involved in fitness calculation, feature selection, or parameter optimization. It is used only after feature selection to independently evaluate the final LSTM-based classification performance. This training-only optimization strategy prevents test-set information from influencing the selected feature subset and minimizes the risk of feature-selection bias.

The PSO parameter settings used to optimize the fused representation are summarized in Table 2. These parameters were selected to achieve an effective balance between search exploration, convergence behavior, and computational efficiency during feature subset optimization. The resulting optimized feature vector is subsequently used as input to the sequential classification stage.

To further validate the effectiveness of the selected optimization strategy, the proposed Particle Swarm Optimization (PSO) approach was compared with Genetic Algorithm (GA) and Grey Wolf Optimization (GWO) using the same experimental settings, and the corresponding performance is summarized in Table 3.

### 3.9. Sequential Modeling Using LSTM

Tumors typically extend across multiple CT slices, and important structural variations may occur along the axial direction. To capture these inter-slice dependencies, an LSTM network is employed to model sequential relationships between slice-wise features. Given a sequence of fused feature vectors extracted from consecutive slices $F=\{f_{1},f_{2},\ldots ,f_{T}\}$ where T denotes the number of slices, the LSTM processes the sequence iteratively using the following gating mechanisms defined in Equations (34)–(39).

Forget gate

(34) $$f_{t}=\sigma \left(W_{f}\left[h_{t-1},x_{t}\right]+b_{f}\right)$$

Input gate

(35) $$i_{t}=\sigma \left(W_{i}\left[h_{t-1},x_{t}\right]+b_{i}\right)$$

Candidate memory

(36) $${\tilde{C}}_{t}=tanh\left(W_{c}\left[h_{t-1},x_{t}\right]+b_{c}\right)$$

Cell state update

(37) $$C_{t}=f_{t}⊙C_{t-1}+i_{t}⊙{\tilde{C}}_{t}$$

Output gate

(38) $$o_{t}=\sigma \left(W_{o}\left[h_{t-1},x_{t}\right]+b_{o}\right)$$

Hidden state

(39) $$h_{t}=o_{t}⊙tanh\left(C_{t}\right)$$

where $x_{t}$ represents the input feature vector at time step t, $h_{t}$ is the hidden state, and ⊙ denotes element-wise multiplication. In this study, a two-layer LSTM network with 128 hidden units in each layer and a dropout rate of 0.3 is applied between the layers to mitigate overfitting. The final hidden representation generated by the LSTM encodes sequential tumor characteristics across slices and is used for the final prediction layer.

#### 3.9.1. Classification Layer

The final classification is performed using a fully connected layer followed by a Softmax activation function. Let $h_{T}$ denote the final hidden state of the LSTM. The predicted probability distribution over K classes is computed as Equation (40).

(40) $$\hat{y}=softmax\left(Wh_{T}+b\right)$$

where W and b represent the learnable parameters of the classification layer. The SoftMax function is defined in Equation (41) as:

(41) $$softmax\left(z_{i}\right)=\frac{e^{z_{i}}}{\sum_{j=1}^{K}e^{z_{j}}}$$

which converts the output scores into class probabilities.

#### 3.9.2. Training Strategy

The proposed model is trained using supervised learning. Given a dataset consisting of N labeled samples represented in Equation (42).

(42) $$\{\left(X_{i},y_{i}\right){\}}_{i=1}^{N}$$

where $X_{i}$ represents the fused feature sequence and $y_{i}$ is the corresponding class label; the model parameters are optimized using the cross-entropy loss function defined in Equation (43) as:

(43) $$L=-\sum_{i=1}^{N}\sum_{k=1}^{K}y_{ik}log({\hat{y}}_{ik})$$

where $y_{ik}$ is the ground truth label and ${\hat{y}}_{ik}$ is the predicted probability for class k. The optimization process iteratively updates network parameters using gradient-based learning to minimize the loss function. The hyperparameter configuration used for training the proposed model is presented in Table 4. The selected settings govern the optimization procedure, learning dynamics, and convergence behavior throughout the training process.

Algorithm 1 summarizes the methodology of the complete framework.
**Algorithm 1** for Lung Cancer Classification Model**Input:** CT volume $V\in R^{S\times H\times W}$, bounding boxes $\left\{B_{s}{\}}_{s=1}^{S}\right.$

**Output:** Predicted histological subtype $\hat{y}$
**1. CT Volume Preprocessing**

**1.1 Intensity clipping:** For each slice s=1…S, $\mathrm{perform}\;\mathrm{lung}\;\mathrm{window}\;\mathrm{intensity}\;\mathrm{clipping}\text{:}\;I_{p}^{s}(x,y)\leftarrow min(\tau_{max},max(\tau_{min},I^{s}(x,y)))$

**1.2 Normalization:**
$\;\mathrm{Normalize}\;\mathrm{clipped}\;\mathrm{slice}\;\mathrm{for}\;\mathrm{model}\;\mathrm{compatibility}\text{:}\;I_{s}^{\prime }(x,y)\leftarrow \frac{I_{p}^{s}(x,y)-min(I_{p}^{s})}{max(I_{p}^{s})-min(I_{p}^{s})}$

**1.3 RGB conversion:**$\;\mathrm{Convert}\;\mathrm{normalized}\;\sin\mathrm{gle}\text{-}\mathrm{channel}\;\mathrm{slice}\;\mathrm{to}\;\mathrm{RGB}\text{:}\;I_{s}^{rgb}(x,y)\leftarrow [I_{s}^{\prime }(x,y),I_{s}^{\prime }(x,y),I_{s}^{\prime }(x,y)]$

**Representation:**
$\;V\leftarrow \{I_{s}^{rgb}{\}}_{s=1}^{S}\in R^{S\times H\times W\times 3}$

**2. Tumor Region Segmentation using MedSAM**

**2.1 Bounding box extraction:**
 Extract bounding box for slice s$\text{:}\;B_{s}\leftarrow (x_{min},y_{min},x_{max},y_{max})$

**2.2 MedSAM mask generation:**
$\;\mathrm{Forward}\;\mathrm{slice}\;\mathrm{and}\;\mathrm{bounding}\;\mathrm{box}\;\mathrm{to}\;\mathrm{MedSAM}\;\mathrm{to}\;\mathrm{generate}\;\mathrm{mask}\text{:}\;M_{s}\leftarrow f_{\mathrm{MedSAM}}(I_{s}^{rgb},B_{s}),M_{s}\in \{0,1{\}}^{H\times W}$

**2.3 Mask merging:**$\;\mathrm{If}\;\mathrm{multiple}\;\mathrm{regions}\;\mathrm{exist},\;\mathrm{merge}\;\mathrm{masks}\;\mathrm{pixel}\text{-}\mathrm{wise}\text{:}\;M_{s}(x,y)\leftarrow {max}_{k}M_{s,k}(x,y)$

**2.4 Tumor slice selection:**$\;\mathrm{Retain}\;\mathrm{tumor}\;\mathrm{slices}\text{:}\;T\leftarrow \{s|\sum_{x,y}M_{s}(x,y)>0\}$
**3. Multi-Level Feature Extraction**
**3.1 Structural and Geometric Features**
**3.1.1 ORB keypoints:** Detect ORB keypoints:
$K_{s}^{ORB}\leftarrow \{(x,y)|I_{p}-I_{i}>\epsilon \;\mathrm{or}\;I_{i}-I_{p}>\epsilon ,\;i\in \{1,\ldots ,16\}\}$ Compute orientation using intensity centroid moments:$\theta \leftarrow {tan}^{-1}\left(\frac{m_{01}}{m_{10}}\right)$ Generate rotation-invariant BRIEF descriptors:$$\tau (p;x,y)\leftarrow \left\{\begin{array}{cc} 1 & \mathrm{if}\;p(x)<p(y)\\ 0 & \mathrm{otherwise} \end{array}\right.$$
**3.1.2 Harris corners:**
$\;\mathrm{Compute}\;\mathrm{Harris}\;\mathrm{corner}\;\mathrm{response}\text{:}\;R(x,y)\leftarrow det(H)-k\cdot (\mathrm{trace}(H){)}^{2},$ $\mathrm{extract}\;\mathrm{corners}\text{:}\;K_{s}^{H}\leftarrow \{(x,y)|R(x,y)>\delta \}$

**3.1.3 Shape descriptors:**$\;\mathrm{Compute}\;\mathrm{spatial}\;\mathrm{moments}\;\mathrm{of}\;\mathrm{mask}\;M_{s}$$\text{:}\;m_{pq}\leftarrow \sum_{x}\sum_{y}x^{p}y^{q}M_{s}(x,y),(\bar{x},\bar{y})=(m_{10}/m_{00},m_{01}/m_{00})$

**Central and normalized moments:**$\;\mu_{pq}\leftarrow \sum_{x}\sum_{y}(x-\bar{x}{)}^{p}(y-\bar{y}{)}^{q}M_{s}(x,y),\eta_{pq}\leftarrow \frac{\mu_{pq}}{\mu_{00}^{1+(p+q)/2}}$

**Hu invariants:**$\;H_{i}^{\prime }\leftarrow -\mathrm{sign}(H_{i})log(|H_{i}|),i=1..7$

**Structural vector:**$\;f_{\mathrm{structure}}^{s}\leftarrow [f_{\mathrm{Hu}}∥K_{s}^{ORB}∥K_{s}^{H}]$
**3.2 Texture Features**

**3.2.1 Quantization:**$\;\mathrm{Quantize}\;\mathrm{intensities}\text{:}\;Q_{s}(x,y)\leftarrow \mathrm{quantize}(I_{s}^{\prime })$
**3.2.2 GLCM computation:** Compute GLCM P(i,j) and derive: Contrast,Homogeneity,Energy,Correlation,Dissimilarity
**3.2.3 LBP computation:**$\;\mathrm{Compute}\;\mathrm{LBP}\text{:}\;\mathrm{LBP}(p)\leftarrow \sum_{n=0}^{P-1}s(g_{n}-g_{c})2^{n},s(x)=1\;\mathrm{if}\;x\geq 0,0\;\mathrm{otherwise}$
**3.2.4 Texture vector:**$\;f_{\mathrm{texture}}^{s}\leftarrow [f_{\mathrm{GLCM}}∥f_{\mathrm{LBP}}]\in R^{d_{t}}$ **3.3 Deep Semantic Features**

**3.3.1 ROI extraction:**$\;\mathrm{Extract}\;\mathrm{tumor}\;\mathrm{ROI}\text{:}\;I_{s}^{roi}\leftarrow I_{s}^{rgb}⊙M_{s}$
**3.3.2 ResNet-50 feature extraction:**$\;\mathrm{Forward}\;\mathrm{through}\;\mathrm{ResNet}\text{-}50,\;\mathrm{global}\;\mathrm{average}\;\mathrm{pooling}\text{:}\;f_{\mathrm{deep}}^{s}\in R^{2048},f_{\mathrm{deep}}^{s}\leftarrow \mathrm{GAP}(F(I_{s}^{roi}))$
**4. Hybrid Feature Fusion**
**4.1 Handcrafted fusion:**$\;f_{\mathrm{hand}}^{s}\leftarrow [f_{\mathrm{structure}}^{s}∥f_{\mathrm{texture}}^{s}]$
**4.2 Hybrid fusion:**$\;f_{\mathrm{hybrid}}^{s}\leftarrow [f_{\mathrm{hand}}^{s}∥f_{\mathrm{deep}}^{s}](\mathrm{dimension}\;d)$
**5. Optimal Feature Selection using PSO**
**5.1 Particle initialization:**$\;\mathrm{Initialize}\;\mathrm{particle}\;\mathrm{population}\;p_{i}\in \{0,1{\}}^{d}$
**5.2 Velocity and position update:**$\;v_{i}\leftarrow wv_{i}+c_{1}r_{1}(pbest_{i}-x_{i})+c_{2}r_{2}(gbest-x_{i}),x_{i}\leftarrow x_{i}+v_{i}$
**5.3 Fitness evaluation and selection:**$\;F\leftarrow \alpha \cdot \mathrm{Acc}-\beta \frac{N_{s}}{N_{f}},f_{\mathrm{opt}}^{s}\leftarrow \mathrm{select}(f_{\mathrm{hybrid}}^{s})$
**6. Sequential Modeling and Classification** **6.1 Feature sequence construction:**$\;F\leftarrow \{f_{\mathrm{opt}}^{1},\ldots ,f_{\mathrm{opt}}^{T}\}$
**6.2 LSTM forward:**$\;h_{t},C_{t}\leftarrow \mathrm{LSTMCell}(x_{t},h_{t-1},C_{t-1})$
**6.3 Final representation:**$\;h_{T}$
**6.4 Logits and SoftMax:**$\;z\leftarrow Wh_{T}+b,{\hat{y}}_{i}\leftarrow \frac{e^{z_{i}}}{\sum_{j}e^{z_{j}}}$
**Return:**$\;\mathrm{Predicted}\;\mathrm{histological}\;\mathrm{subtype}\;\hat{y}$


## 4. Experimental Results

### 4.1. Dataset Description 

The proposed framework was evaluated using two publicly available lung CT datasets: Lung-PET-CT-Dx and LIDC-IDRI. The Lung-PET-CT-Dx dataset, curated by The Cancer Imaging Archive (TCIA), is a large-scale repository containing paired anatomical CT and molecular PET images in standard DICOM format. Tumor localization was performed by five thoracic radiologists with more than three years of clinical experience, and the annotations were provided as bounding boxes in XML (PASCAL VOC) format to facilitate lesion localization and subsequent segmentation. Histopathological findings obtained from lung biopsies serve as the prompt, enabling reliable classification into four major malignant subtypes: Adenocarcinoma (ADC), Small Cell Carcinoma (SCC), Large Cell Carcinoma (LCC), and Squamous Cell Carcinoma (SQC). Due to the very limited number of original patient cases in the LCC category, LCC was excluded from classification experiments to ensure a more reliable evaluation. To mitigate class imbalance, data augmentation was applied.

To further assess the robustness and generalization capability of the proposed framework, experiments were also conducted on the LIDC-IDRI dataset, a widely used multi-institutional public repository available through TCIA. The dataset contains thoracic CT scans collected from multiple clinical centers with nodule annotations provided independently by up to four experienced thoracic radiologists using a two-phase annotation protocol. Based on the assigned malignancy ratings, the nodules were categorized into Benign (BN), Indeterminate (ID), and Malignant (MG) classes for classification [22]. The diversity of imaging characteristics, expert annotations, and acquisition protocols makes LIDC-IDRI a valuable benchmark for assessing the generalizability of the proposed framework. The class-wise distribution of samples for both datasets is presented in Table 5.

### 4.2. Experimental Protocol and Assessment Matrices

The proposed framework was evaluated using 5-fold cross-validation to ensure robust and unbiased performance assessment. The dataset was randomly divided into five equal subsets, where in each iteration, 80% of the data was used for training and 20% for testing. The process was repeated five times, with each subset serving as the test set once, and the final results were obtained by averaging the performance across all folds. To prevent data leakage, dataset partitioning was performed at the patient/CT-volume level before slice extraction. All slices belonging to a particular patient were exclusively assigned to either the training or testing subset, ensuring that no slices from the same patient occurred across the two subsets.

The performance of the proposed framework was evaluated using several widely adopted classification metrics to provide a comprehensive assessment of prediction quality across all lung cancer subtypes. These metrics quantify different aspects of model performance, including overall correctness, class-wise prediction reliability, detection capability, and agreement between predicted and actual labels. Classification accuracy measures the proportion of correctly classified samples among all evaluated instances and is computed as Equation (44).

(44) $$\mathrm{Accuracy}=\frac{TP+TN}{TP+TN+FP+FN}$$

where TP, TN, FP, and FN denote the numbers of true positives, true negatives, false positives, and false negatives, respectively. Precision evaluates the reliability of positive predictions by measuring the fraction of predicted positive samples that are correctly classified. It is defined as Equation (45).

(45) $$\mathrm{Precision}=\frac{TP}{TP+FP}$$

Recall, also referred to as sensitivity, measures the ability of the model to correctly identify positive instances and is given by Equation (46).

(46) $$\mathrm{Recall}=\frac{TP}{TP+FN}$$

To provide a balanced assessment of precision and recall, the F1-score is computed in Equation (47) as the harmonic mean of the two measures:

(47) $$F_{1}=2\times \frac{\mathrm{Precision}\times \mathrm{Recall}}{\mathrm{Precision}+\mathrm{Recall}}$$

In addition to these metrics, Cohen’s Kappa coefficient is used to evaluate the level of agreement between predicted and actual class labels while accounting for agreement occurring by chance. It is calculated as Equation (48).

(48) $$\kappa =\frac{p_{o}-p_{e}}{1-p_{e}}$$

where $p_{o}$ represents the observed agreement and $p_{e}$ denotes the expected agreement by random chance.

The discriminative capability of the proposed model is further assessed using the Area Under the Receiver Operating Characteristic Curve (AUC-ROC). The ROC curve is generated by plotting the True Positive Rate (TPR) against the False Positive Rate (FPR) at different classification thresholds. These measures are defined as Equations (49) and (50):

(49) $$TPR=\frac{TP}{TP+FN}$$

(50) $$FPR=\frac{FP}{FP+TN}$$

A higher AUC value indicates stronger class separability and improved classification performance. Collectively, these metrics provide a comprehensive evaluation of the proposed framework and enable reliable comparison with existing state-of-the-art methods.

### 4.3. Confusion Matrix and Performance Evaluation

The confusion matrices presented in Table 6 correspond to the single run among five independent training runs using the same experimental configuration. For each dataset, the original samples were randomly partitioned into 80% training and 20% testing subsets, with augmentation applied exclusively to the training set after patient-level data splitting. The revised evaluation includes three classification settings: three-class classification on Lung-PET-CT-Dx [23,24,25,26], binary classification experiment on LIDC-IDRI excluding the ID class [27,28,29,30,31,32] and an additional three-class classification on LIDC-IDRI [33,34]. For the Lung-PET-CT-Dx dataset, the confusion matrix shows correct classification of 32 ADC, 16 SQC, and 13 SCC samples, with relatively few misclassifications among the three histological subtypes. For the three-class LIDC-IDRI evaluation, the model correctly classified 325 BN, 102 ID, and 332 MG samples, with the ID class showing comparatively greater confusion with the other categories. To further evaluate the model under a reduced-class setting, a binary LIDC-IDRI experiment was additionally conducted by excluding the ID class. In this setting, 329 BN and 332 MG samples were correctly classified, with relatively few errors between the two classes. Overall, the confusion matrices provide detailed class-wise evidence of the proposed framework’s performance across the different classification settings, while the additional binary experiment further evaluates discrimination between benign and malignant cases under a simplified clinical classification scenario.

The quantitative evaluation presented in Table 7 demonstrates the consistent performance of the proposed framework across the evaluated classification settings. For the Lung-PET-CT-Dx dataset, the model achieved a Precision of 0.92 ± 0.024 (95% CI: 0.873–0.967), Recall of 0.92 ± 0.025 (95% CI: 0.871–0.969), and F1-score of 0.92 ± 0.022 (95% CI: 0.876–0.964), with an overall Cohen’s Kappa of 0.9150, indicating strong agreement between the predicted and actual classes. For the three-class LIDC-IDRI classification, the model obtained a Precision of 0.90 ± 0.028 (95% CI: 0.845–0.955), Recall of 0.89 ± 0.031 (95% CI: 0.829–0.951), and F1-score of 0.895 ± 0.025 (95% CI: 0.846–0.944), with a Cohen’s Kappa of 0.8891. In the additional binary LIDC-IDRI experiment, excluding the ID class, the framework achieved a Precision of 0.93 ± 0.024 (95% CI: 0.90–0.96), Recall of 0.93 ± 0.028 (95% CI: 0.90–0.96), and F1-score of 0.93 ± 0.021 (95% CI: 0.90–0.96), with a Cohen’s Kappa of 0.8566.

### 4.4. Comparison with Existing Work

The comparative analysis presented in Table 8 demonstrates the competitive performance of the proposed framework across the evaluated lung CT classification settings. For the Lung-PET-CT-Dx dataset, the proposed method achieves an accuracy of 93.70%, outperforming several existing approaches, including Loja et al. [17] (78.00%), Huang et al. [23] (90.32%), Hahn et al. [24] (89.98%), %), Dunn et al. [15] (92.70%) Chowdhury et al. [18] (91.36%), , Barbouchi et al. [19] (74.00% and 78.00%), Zeng et al. [20] (90.70%), Jacob et al. [25] (93.10%), and Abdulqader et al. [26] (92.00%). On the LIDC-IDRI dataset, the proposed framework achieves 94.70%accuracy in the binary classification setting, exceeding the performance reported by Sun et al. [27] (90.60%), Shuvo et al. [28] (93.57%), Al Duhayyim et al. [29] (88.30% and 94.10%), Huang et al. [30] (91.07%), Wu et al. [31] (92.36%), and Gupta et al. [32] (91.30%) in the corresponding reported settings. Furthermore, the proposed framework achieves 92.90%accuracy for the three class classification setting, compared with 85.88% and 84.81% reported by Lyu et al. [33,34], respectively. Overall, these results demonstrate the competitive generalization capability of the proposed framework across different datasets and classification settings. The performance is attributed to the integration of MedSAM-guided tumor delineation, hybrid handcrafted and deep feature representation, PSO-based feature optimization, and LSTM-based sequential modeling.

### 4.5. ROC Curves

**Figure 7:**
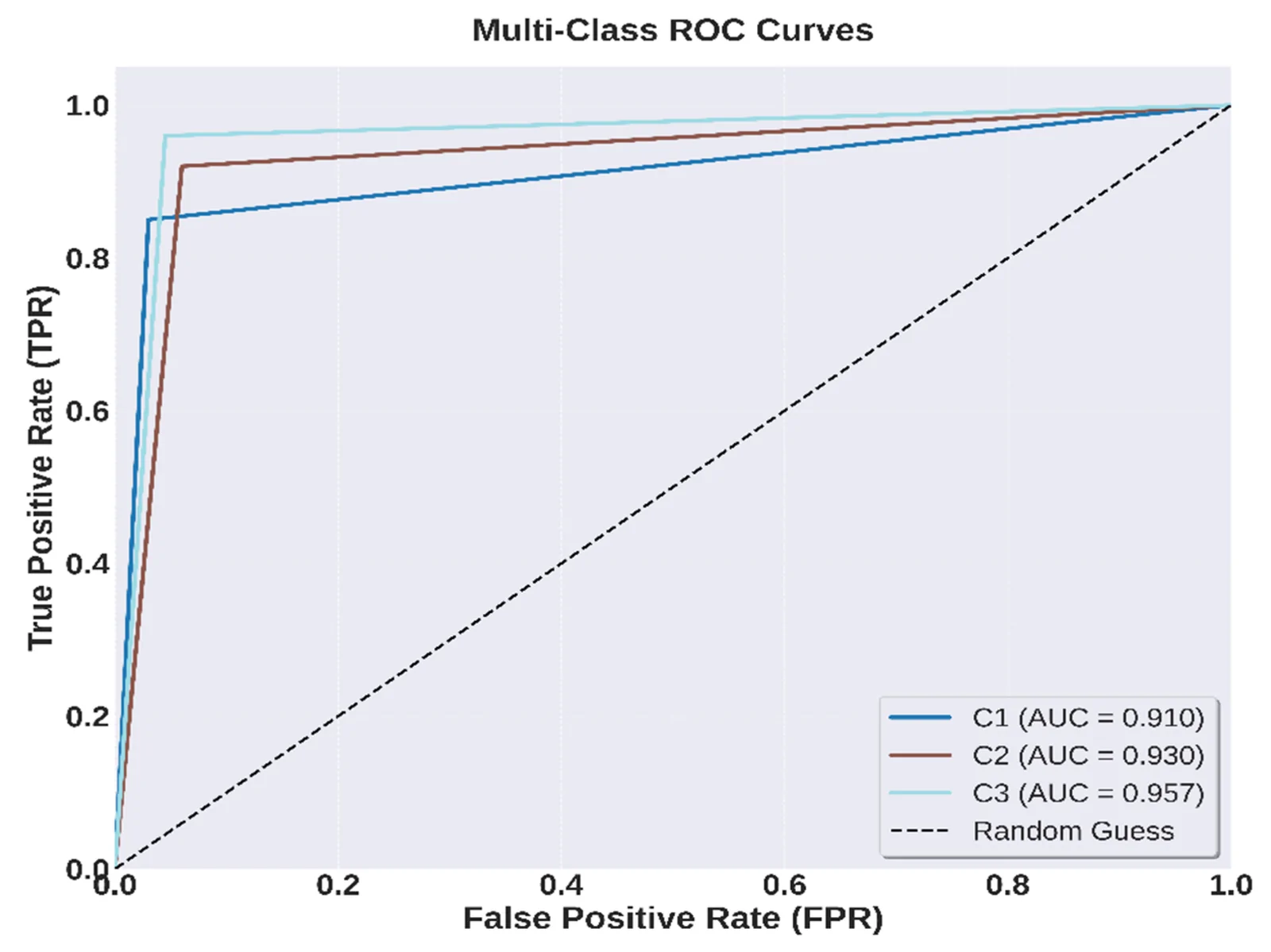
ROC Curves of Lung-PET-CT-Dx dataset. C1 =ADC, C2 = SQC, C3 = SCC.

Figure 7 shows the ROC curve of the proposed model on the Lung-PET-CT-Dx dataset when it is used to do multi-class classification. The curve shows that the model is highly effective in distinguishing among the various subtypes of lung cancer as indicated by its closeness to the upper-left hand corner. The achieved Area Under the Curve (AUC) of about 0.95 shows that it has good discriminative ability with all classes. The large AUC value is in line with the confusion matrix results, most of which have high true positive rates, especially SCC (0.957).

Even though some difficulties with misclassification are noted in some of the classes like the SCC, the overall ROC behavior proves that the model has a good balance between sensitivity and specificity. Moreover, the ROC analysis corresponds to the general performance indicators, such as accuracy of 93.7% and Cohen Kappa of 0.915 which indicates a high degree of agreement and the strongness of the suggested framework. The proposed model exhibits a higher classification potential than current State-of-the-Art techniques, which supports the notion that it can be effectively used to predict lung cancer subtypes with high reliability.

**Figure 8:**
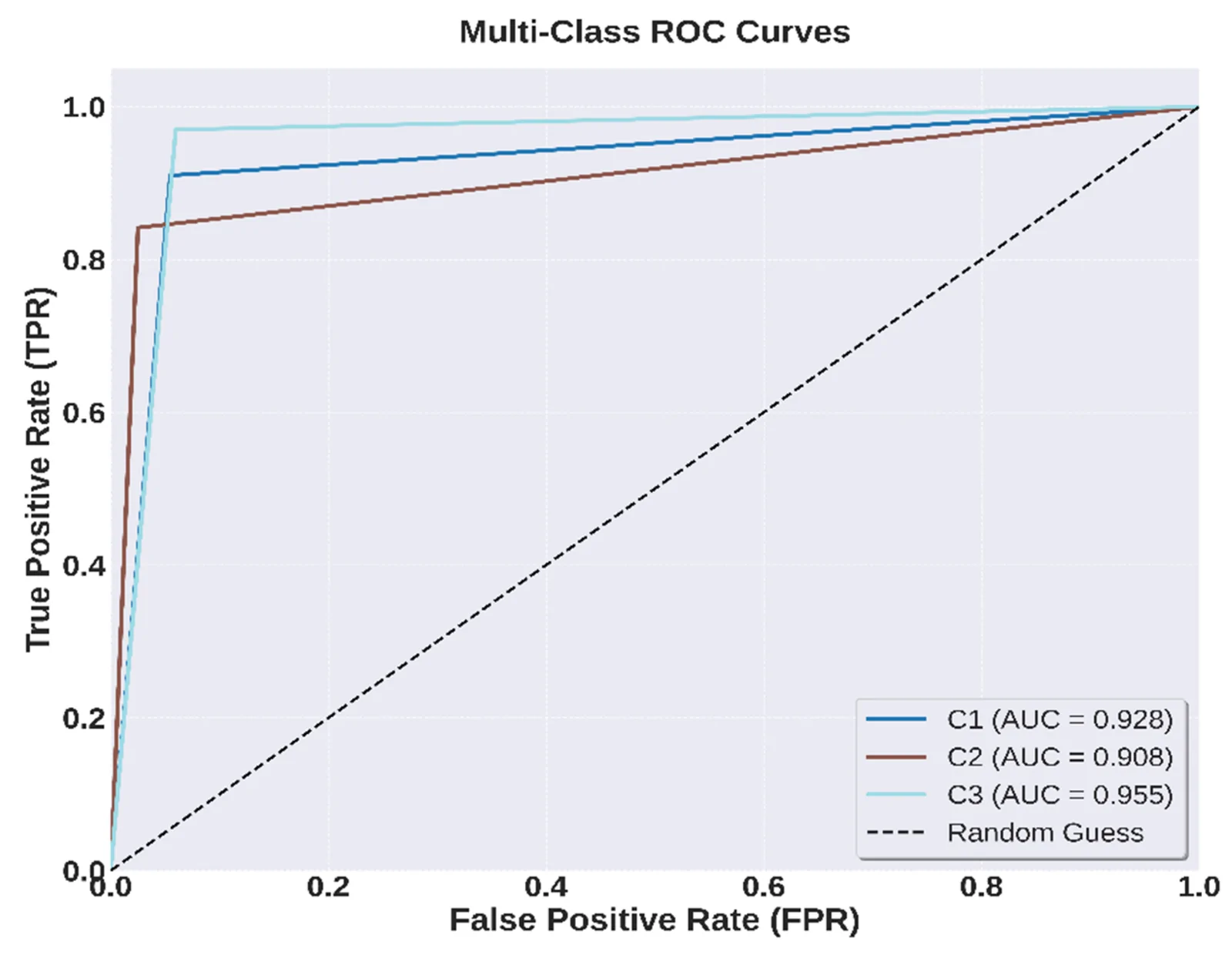
ROC Curves of LIDC-IDRI dataset. C1 = BN, C2 = ID, C3 = MG.

Figure 8 presents the ROC curves of the proposed framework on the LIDC-IDRI dataset for three-class classification. The curves remain close to the upper-left corner, indicating strong discrimination among BN, ID, and MG classes. The obtained AUC values of 0.92, 0.90, and 0.95, respectively, demonstrate consistently high classification performance, with the slightly lower AUC for ID reflecting its greater overlap with the other two classes. Overall, the ROC analysis supports the achieved classification accuracy of 94.7% and confirms the robustness of the proposed framework for pulmonary nodule classification.

**Figure 9:**
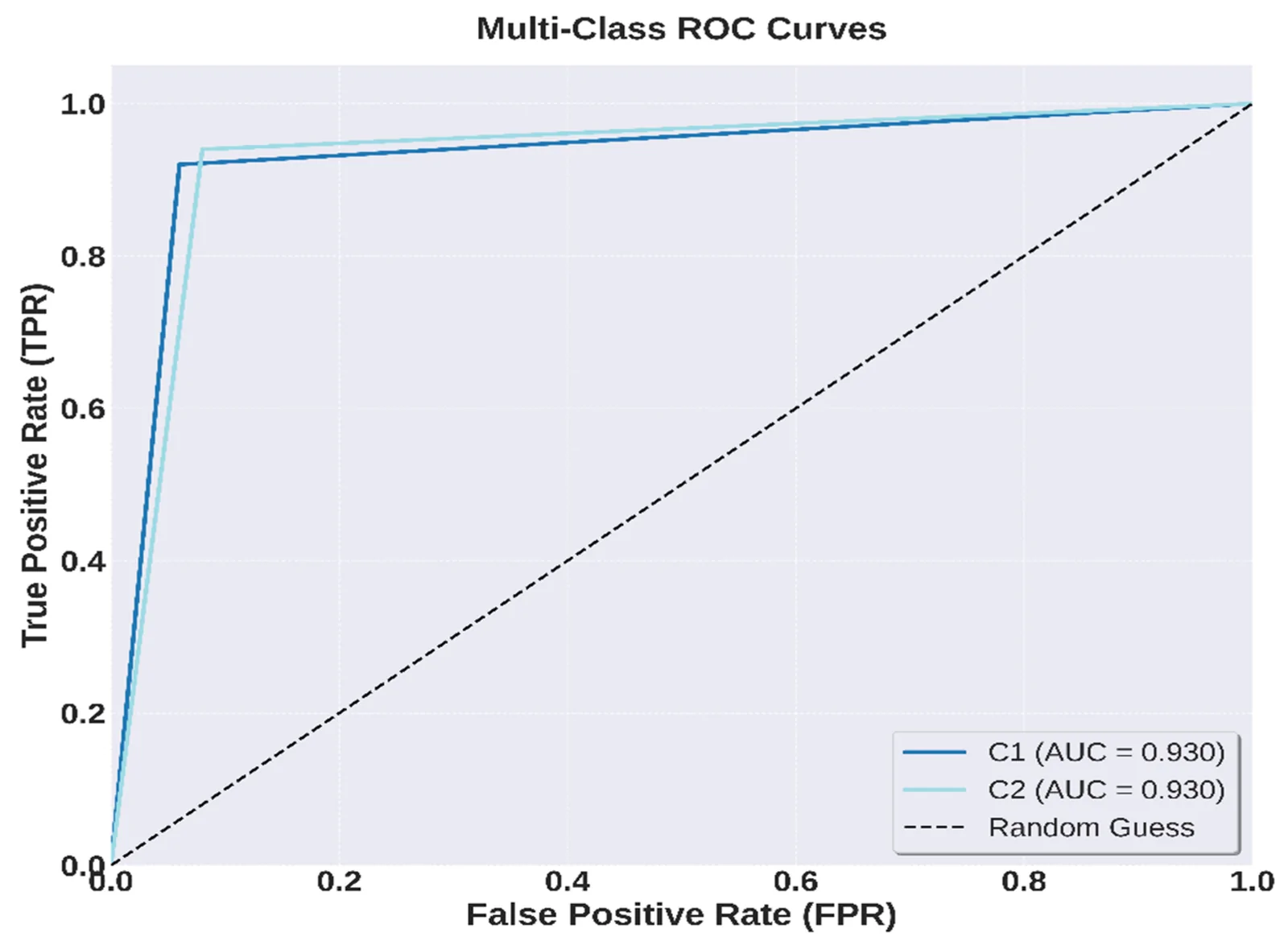
ROC Curves of LIDC-IDRI dataset for binary classification. C1 = BN, C2 = MG.

The ROC analysis for the binary classification experiment on the LIDC-IDRI dataset is shown in Figure 9. The model achieved an AUC of 0.930 for both BN and MG classes, indicating strong discriminatory capability between benign and malignant nodules. The closely overlapping class-wise ROC curves further demonstrate consistent classification performance across the two categories.

### 4.6. Ablation Study

Table 9 presents the ablation study conducted to evaluate the contribution of each component in the proposed lung cancer classification framework on the Lung-PET-CT-Dx dataset. The complete model, which integrates preprocessing, deep feature extraction using ResNet-50, structural keypoints, shape descriptors, textural features, and feature optimization via PSO, achieves the highest accuracy of 93.70%, demonstrating the effectiveness of the hybrid feature fusion strategy.

When preprocessing is removed, the accuracy drops to 88.40%, highlighting the importance of intensity clipping, volume standardization and normalization in improving data quality and consistency. The most significant performance degradation is observed when deep features are excluded, with accuracy falling sharply to 69.10%, indicating that high-level semantic representations extracted from ResNet-50 are critical for effective classification of lung cancer subtypes.

Similarly, removing structural keypoints (Harris and ORB) reduces the accuracy to 84.10%, confirming that local structural information contributes meaningfully to capturing discriminative patterns. Excluding shape descriptors (Hu Moments) results in an accuracy of 88.30%, demonstrating that morphological characteristics play a supportive role in classification. Furthermore, omitting textural features (LBP and GLCM) leads to a decrease in accuracy to 82.70%, emphasizing the importance of texture information in differentiating between tumor types. Finally, the removal of feature optimization using PSO reduces the accuracy to 76.30%, indicating that optimization plays a crucial role in selecting the most relevant features and improving overall model performance.

To further extend the ablation study, a comparative analysis was conducted using different temporal and conventional classifiers to evaluate their contribution to the overall classification performance. As shown in Table 10, the proposed LSTM achieved a classification accuracy of 93.70%, demonstrating highly competitive performance among the evaluated deep learning classifiers. The Transformer achieved a slightly lower accuracy of 92.90%, while GRU, RNN, TCN, GNN, and MLP achieved 91.10%, 91.90%, 87.50%, 87.70%, and 84.20%, respectively. The close performance of LSTM and Transformer indicates that LSTM provides a strong predictive capability while offering a simpler sequential modeling mechanism. In addition, conventional classifiers, including SVM, Random Forest, Naive Bayes, and XGBoost, were evaluated to provide a broader comparison of classification strategies. Overall, the results support the selection of LSTM as an effective classification component within the proposed framework, providing a strong balance between classification performance and model simplicity.

Although the ResNet-50 branch provides the largest contribution to predictive performance, its learned representations are not inherently interpretable. Therefore, Grad-CAM was employed to provide a visual explanation of the deep model by identifying the spatial regions that contribute most strongly to its prediction. This complements the handcrafted descriptors, which provide explicit numerical representations of tumor structure, shape, and texture. Consequently, interpretability in the proposed framework is achieved through both feature-level characterization and visual explanation of the deep representation.

Overall, the ablation results clearly demonstrate that each component contributes positively to the final performance, with deep feature extraction and preprocessing having the most significant impact. The combination of multi-level features and optimization enables the proposed framework to achieve superior accuracy compared to reduced configurations.

### 4.7. Explainability Analysis Using Grad-CAM

To qualitatively interpret the decision-making behavior of the deep learning branch, Gradient-weighted Class Activation Mapping (Grad-CAM) was employed to visualize the spatial regions contributing most significantly to the model’s predictions. Grad-CAM generates class-discriminative localization maps by utilizing the gradients of the predicted class with respect to the intermediate convolutional feature maps, thereby highlighting anatomically relevant regions within the CT images. This visualization provides an intuitive explanation of the learned representations without modifying the trained network.

**Figure 10:**
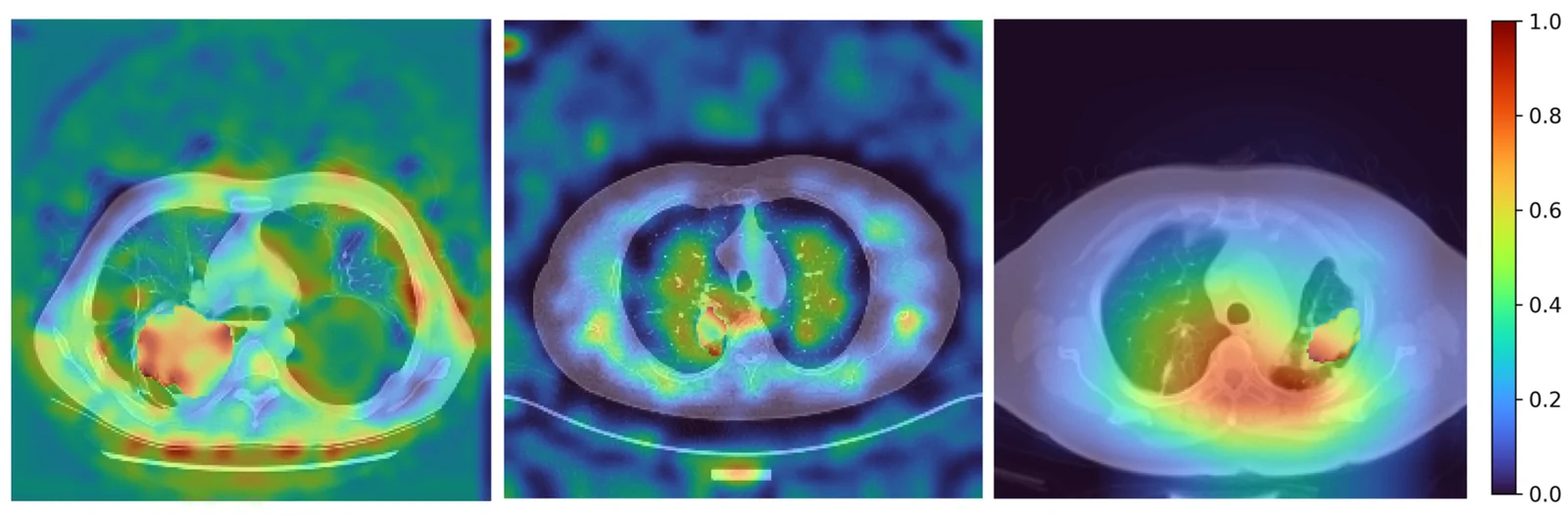
Representative Grad-CAM maps of the ResNet-50 backbone illustrating localization of discriminative regions during deep feature extraction.

Figure 10 presents Grad-CAM visualizations obtained from multiple convolutional stages of the deep feature extraction network. The early layers primarily respond to low-level anatomical structures such as tissue boundaries and intensity transitions, whereas progressively deeper layers exhibit increasingly localized attention around the tumor region while suppressing surrounding healthy lung parenchyma. The deepest convolutional features produce the most discriminative activation maps, indicating that the network successfully learns high-level semantic representations associated with lung tumor morphology.

### 4.8. Computational Analysis

In addition to performance evaluation, a computational analysis was conducted to assess the efficiency and complexity of each component in the proposed framework. As shown in Table 11, the results indicate that most handcrafted feature extraction techniques, including GLCM, LBP, Hu Moments, and keypoint detectors (ORB and Harris), incur minimal computational overhead, with very low GFLOPs and inference times. In contrast, the segmentation module (MEDSAM) contributes the highest computational cost (488.24 GFLOPs), making it the most resource-intensive stage of the pipeline. The deep feature extractor, ResNet-50, demonstrates a moderate computational requirement (4.11 GFLOPs) while providing significant performance gains, as evidenced in the ablation study. Preprocessing operations are computationally negligible but play a crucial role in improving model accuracy. Overall, the analysis highlights that the proposed framework achieves a favorable balance between classification performance and computational efficiency, where high-cost components such as MEDSAM and ResNet-50 are justified by their substantial contribution to predictive accuracy, while the remaining modules add discriminative power with minimal additional cost.

## 5. Limitations

This study has several limitations that should be acknowledged. The proposed framework was developed and validated using a retrospective dataset, which may limit its generalizability to diverse patient populations, scanner types, and imaging protocols across different institutions. Although multiple feature types were integrated, the model’s performance might be affected by variations in tumor size, location, and imaging artifacts commonly encountered in routine clinical practice. Furthermore, although prompt-based MedSAM segmentation reduces the need for pixel-level manual annotation, the current implementation relies on externally provided spatial prompts, which may introduce user dependency and variability and limit fully automated deployment. Future work will therefore investigate self-prompting and automated prompt-generation strategies to reduce manual intervention and improve the consistency and scalability of tumor delineation. Additionally, MedSAM was deployed in a zero-shot manner without retraining or fine-tuning on the utilized dataset, which may limit segmentation accuracy for domain-specific imaging characteristics. Finally, while promising results were achieved for histological subtype differentiation, the relatively limited number of cases for certain rare subtypes may affect the robustness of the findings. Future work will focus on addressing these challenges through quantitative segmentation evaluation and refinement and prospective clinical evaluation.

## 6. Conclusions

The increasing global burden of lung cancer highlights the need for accurate and clinically reliable AI-driven diagnostic systems to support early detection and clinical decision-making. To address challenges arising from tumor heterogeneity and variability in CT interpretation, this study proposed an AI-based framework integrating MedSAM-driven tumor segmentation, radiomic biomarkers, and deep feature learning for lung cancer characterization and histological subtype classification. By combining interpretable radiomic descriptors with deep semantic representations, the proposed approach enables comprehensive tumor characterization while preserving clinical interpretability. Feature fusion and optimization further reduce redundancy in high-dimensional spaces, improving robustness and generalization across diverse imaging conditions. Experimental results show that the framework achieves a peak accuracy of 95.4% across 12 benchmark comparisons, outperforming existing methods and demonstrating the effectiveness of segmentation-guided, radiomics-enhanced deep learning for diagnostic analysis. Overall, the proposed model supports AI-assisted lung cancer diagnosis and strengthens clinical decision support, with future work focusing on quantitative segmentation evaluation and multimodal integration to enhance real-world clinical applicability.

## Figures and Tables

**Table 1:** Extracted Feature dimensions and fused feature vector dimension.

Feature	Feature Vector Dimension
ORB	3200 (e.g., 100 keypoints × 32)
Harris	200 (e.g., 100 keypoints × 2D)
Hu Moments	7
GLCM	5
LBP	10
Resnet-50	2048
Total	5470

**Table 2:** Parameter settings used for PSO.

Parameter	Value
Number of particles (swarm size)	40
Number of iterations	150
Inertia weight (w)	0.7
Cognitive coefficient ($c_{1}$)	2.0
Social coefficient ($c_{2}$)	2.0
α (accuracy weight)	0.99
β (sparsity weight)	0.01

**Table 3:** Comparison of PSO with other algorithms on Lung-PET-CT-Dx dataset.

Optimization Algorithm	PSO	GWO	GA
Accuracy	93.70	88.10%	91.70%

**Table 4:** Training hyperparameters for the proposed framework.

Hyperparameter	Value
Number of epochs	100
Batch size	32
Optimizer	Adam
Learning rate	0.001
Loss function	Categorical Cross-Entropy
Early Patience	10
Random seed	42

**Table 5:** Class-wise distribution of samples for both datasets.

Dataset	Classes	Number of Samples
Lung-PET-CT-Dx	ADC	251
	SQC	61
	SCC	38
	LCC	5
LIDC-IDRI	BN	1384
	ID	722
	MG	1989

**Table 6:** Confusion Matrix Analysis of Proposed Framework.

Confusion Matrix Analysis for Proposed Work for LUNG-PET-CT-Dx Dataset				Confusion Matrix Analysis for Proposed Work for LIDC-IDRI Dataset (3 Classes)				Confusion Matrix Analysis for Proposed Work for LIDC-IDRI Dataset (Binary Classification)		
True/Pred	ADC	SQC	SCC	True/Pred	BN	ID	MG	True/Pred	BN	MG
**ADC**	32	4	2	**BN**	325	14	18	**BN**	329	29
**SQC**	1	16	1	**ID**	9	102	9	**MG**	19	297
**SCC**	0	1	13	**MG**	7	3	332	-	-	-

**Table 7:** Performance evaluation of proposed work.

Performance Evaluation of Proposed Work				
Dataset	Precision	Recall	F1-Score	Kappa (Overall)
Lung-PET-CT-Dx	0.92 ± 0.024 (95% CI: 0.873–0.967)	0.92 ± 0.025 (95% CI: 0.871–0.969)	0.92 ± 0.022 (95% CI: 0.876–0.964)	0.9150
LIDC-IDRI (three class classification	0.90 ± 0.028 (95% CI: 0.845–0.955)	0.89 ± 0.031 (95% CI: 0.829–0.951)	0.895 ± 0.025 (95% CI: 0.846–0.944)	0.8891
LIDC-IDRI (binary classification)	0.93 ± 0.024 (95% CI: 0.90–0.96)	0.93 ± 0.028 (95% CI: 0.90–0.96)	0.93 ± 0.021 (95% CI: 0.90–0.96)	0.8566

**Table 8:** Comparison of proposed framework with State-of-the-Art models.

Datasets	Author (s)	Year	Accuracy
Lung-PET-CT-Dx	Dunn et al. [15]	2023	92.70%
	Wang et al. [16]	2020	81.00%
	Loja et al. [17]	2025	78.00%
	Chowdhury et al. [18]	2025	91.36%
	Barbouchi et al. [19]	2023	78.00%
	Barbouchi et al. [19]	2023	74.00%
	Zeng et al. [20]	2025	90.70%
	Huang et al. [23]	2024	90.32%
	Hahn et al. [24]	2025	89.98%
	Jacob et al. [25]	2021	93.10%
	Abdulqader et al. [26]	2025	92.00%
	Proposed Method		93.70%
LIDC-IDRI	Sun et al. [27]	2024	90.60%
	Shuvo et al. [28]	2026	93.57%
	Al Duhayyim et al. [29]	2026	88.30%
	Al Duhayyim et al. [29]	2026	94.10%
	Huang et al. [30]	2022	91.07%
	Wu et al. [31]	2023	92.36%
	Gupta et al. [32]	2024	91.30%
	Proposed Method (binary classification)		94.70%
	Lyu et al. [33]	2020	85.88%
	Lyu et al. [34]	2018	84.81%
	Proposed Method (three class classification)		92.90%

**Table 9:** Ablation study on model configuration for lung cancer classification.

Ablation Analysis for Proposed Work		
Model Configuration	Description	Accuracy
All Parameters	Model trained using all feature extraction techniques, including preprocessing, deep features extraction, structural keypoints, shape descriptors, textural features)	93.70%
Without Preprocessing	Model trained without image preprocessing (intensity clipping, volume standardization, intensity normalization).	88.40%
Without RESNET-50	Model trained without Deep features (Resnet 50)	69.10%
Without Structural Keypoints	Model trained without Harris keypoints and ORB	84.10%
Without Shape descriptors	Model trained without hu moments	88.30%
Without Textural Features	Model trained without (LBP, GLCM)	82.70%
Without Feature optimization	Model trained without Particle Swarm Optimization	76.30%

**Table 10:** Comparison LSTM with other classifiers.

Classifier	LSTM	RNN	MLP	GNN	Transformer	TCN	GRU
Accuracy	93.70	90.10	84.20	87.70	92.90	87.50	91.10
Classifier	**SVM**		**RF**		**NB**		**XGBoost**
Accuracy	84.90		86.1		78.8		80.20

**Table 11:** Computational analysis on model configuration for lung cancer classification.

Computational Analysis for Proposed Work		
Method	Inference Time (s)	Complexity (GFLOPs)
Preprocessing	0.00344	0.001006
MEDSAM	0.00752	488.24
GLCM	0.07890	0.000335
LBP	0.04690	0.002097
Hu Moments	0.0003734	0.001311
ORB	0.01509	0.00655
HARRIS	0.01509	0.00655
ResNet50	0.02387	4.11

## Data Availability

The dataset used in this study was Lung-PET-CT-Dx and is available online at https://www.cancerimagingarchive.net/collection/lung-pet-ct-dx/ (accessed on 9 June 2026). The dataset was collected by the Computer Center and Cancer Institute at the Second Affiliated Hospital of Harbin Medical University, China for lung cancer research. The dataset contains PET and CT scans of more than 355 patients in DICOM format along with associated clinical and diagnostic information. It is widely used in medical imaging, tumor segmentation and deep learning-based lung cancer diagnosis and prognosis studies due to its multimodal imaging characteristics and publicly accessible standardized data. The second dataset used in this study was LIDC-IDRI (Lung Image Database Consortium and Image Database Resource Initiative), which is publicly available through The Cancer Imaging Archive (TCIA) at https://www.cancerimagingarchive.net/collection/lidc-idri/ (accessed on 9 June 2026). It contains thoracic CT scans from 1018 patients in DICOM format with expert-annotated lung nodules and associated diagnostic information. Due to its high-quality annotations and standardized imaging data, LIDC-IDRI is widely used for lung nodule detection, segmentation, classification, and deep learning-based lung cancer research.
